# Translational evaluation of gait behavior in rodent models of arthritic disorders with the CatWalk device – a narrative review

**DOI:** 10.3389/fmed.2023.1255215

**Published:** 2023-10-06

**Authors:** Jana Ritter, Maximilian Menger, Steven C. Herath, Tina Histing, Jonas Kolbenschlag, Adrien Daigeler, Johannes C. Heinzel, Cosima Prahm

**Affiliations:** ^1^Department of Hand-, Plastic, Reconstructive and Burn Surgery, BG Klinik Tuebingen, University of Tuebingen, Tuebingen, Germany; ^2^Department of Trauma and Reconstructive Surgery, BG Klinik Tuebingen, University of Tuebingen, Tuebingen, Germany; ^3^Ludwig Boltzmann Institute for Traumatology – The Research Center in Cooperation with AUVA, Vienna, Austria; ^4^Austrian Cluster for Tissue Regeneration, Vienna, Austria

**Keywords:** CatWalk, gait analysis, arthritic disorders, pain behavior, rodents, Osteoarthritis, Monoarthritis, Rheumatoid Arthritis

## Abstract

Arthritic disorders have become one of the main contributors to the global burden of disease. Today, they are one of the leading causes of chronic pain and disability worldwide. Current therapies are incapable of treating pain sufficiently and preventing disease progression. The lack of understanding basic mechanisms underlying the initiation, maintenance and progression of arthritic disorders and related symptoms represent the major obstacle in the search for adequate treatments. For a long time, histological evaluation of joint pathology was the predominant outcome parameter in preclinical arthritis models. Nevertheless, quantification of pain and functional limitations analogs to arthritis related symptoms in humans is essential to enable bench to bedside translation and to evaluate the effectiveness of new treatment strategies. As the experience of pain and functional deficits are often associated with altered gait behavior, in the last decades, automated gait analysis has become a well-established tool for the quantitative evaluation of the sequalae of arthritic disorders in animal models. The purpose of this review is to provide a detailed overview on the current literature on the use of the CatWalk gait analysis system in rodent models of arthritic disorders, e.g., Osteoarthritis, Monoarthritis and Rheumatoid Arthritis. Special focus is put on the assessment and monitoring of pain-related behavior during the course of the disease. The capability of evaluating the effect of distinct treatment strategies and the future potential for the application of the CatWalk in rodent models of arthritic disorders is also addressed in this review. Finally, we discuss important consideration and provide recommendations on the use of the CatWalk in preclinical models of arthritic diseases.

## 1. Introduction

The term “arthritic disorders” refers to >100 different entities ranging from acute joint inflammation to polyarthritic rheumatological disorders. Osteoarthritis (OA) is the most prevalent arthritic disorder, mainly affecting the knee, followed by the hand and hip (1). In 2019, 344 million people worldwide suffered from OA, while 13 million were diagnosed with Rheumatoid Arthritis (RA). Years of life lived with disability attributable to OA and RA have increased by over 100% from 1990 to 2019 (2).

OA is one of the most common reasons for consulting a medical professional, while pain associated with OA is the mean reason for treatment initiation (3, 4). Pain is the major complaint of OA patients, followed by joint stiffness and functional impairment (5–8). However, despite recent advances in preclinical arthritis research, mechanisms underlying pain and functional impairment are poorly understood and sufficient treatment remains challenging. Current therapy approaches often do not provide adequate pain relief and improvement of locomotor function, lack long- term efficacy and are questioned with regard to their long-term safety and development of adverse effects (9). Moreover, in the past decade several studies have pointed to the discrepancy between radiological evident signs of OA and clinical presentation of symptomatic OA (6, 10–16). In the context of inadequate therapies, chronic, persistent pain, disability and lack of correlation between patients’ complaints and radiographic evidence of OA, it seems surprising that in preclinical research, histological changes continue to be the primary endpoints. Outcome measures not only need to mimic human OA in terms of pathology but also resemble the clinical presentation of OA. This reflects the need to incorporate functional test into preclinical models of arthritic disorders enabling measurement of clinically relevant endpoints and quantification of behavioral responses considered equivalent to pain and disability in humans. In correlation with histological examinations and imaging modalities this may reveal new insights into the processes and mechanisms underlying the development of OA related pain and disability.

Although a myriad of behavioral tests have been established in animal models to evaluate pain and function, their validity, reliability, and clinical relevance have been questioned as most of them are stimulus-evoked, experimenter dependent and involve stressful manipulation of the animals (17). However, in recent years gait analysis has been claimed as valuable tool in the analysis of arthritic disorders in both clinical and preclinical research. Over time, gait analysis evolved from observational scoring and the analysis of ink prints on a paper over video analysis of gait to the development of computerized and fully-automated gait analysis systems such as the DigiGait, TreadScan and the CatWalk (CW). These systems now allow for multidimensional analysis of spatial and temporal dynamics of gait and locomotion (18–21). Changes of locomotion are important consequences of musculoskeletal disorders and numerous preclinical studies have revealed that gait behavior is an indirect indicator of pain (22–25). Gait analysis appears to be a clinically relevant outcome, as the evaluation of pain and functional impairment during walking is consistent with the complaints experienced by patients with OA (26).

Of note the CW is only one of several commercially available devices to study gait in rodents with arthritic disorders, and several studies using alternatives such as the DigiGait (Mouse Specifics, Inc., Framingham, Massachusetts, United States), TreadScan and GaitScan (CleverSys Inc., Reston, Virginia, United States) have been published (27). Since all these systems are more or less different from each other either regarding the technology used to visualize the animal’s paw prints or regarding the specifications of the used hardware, outcomes are difficult to compare and it has been shown that these systems do not necessarily achieve the same results (27). Given the fact that the CW system is the most commonly used and cited system to evaluate gait in rodent models of arthritic disorders and this method’s advantages such as illuminating the portion of the foot touching the floor and automated classification of the footprints, we decided to focus on this device. Nevertheless, the commercially available aforementioned devices also have unique advantages such as treadmill-controlled fixed speed (DigiGait) (28) or the ability to capture lateral and ventral views of the animal (TreadScan) (27) which might be particular useful analyzes depending from the specific model and research question investigated.

The CW was originally developed by Frank Hamers in 2001 with the intention to evaluate gait in rodents with spinal cord injury (29). Since, the CW has been validated in numerous models of central and peripheral nervous as well as musculoskeletal injuries (27, 30, 31). The system consists of an enclosed walkway with a glass floor of 0.6 cm thickness which is illuminated by green LED lights floor, while red light is emitted from the ceiling of the CW. With no animal on the glass walkway the light is completely reflected. When rodents voluntarily traverse the walkway, the green light scatters at those areas where the paws or other parts of the body are in contact with the floor and illuminates these areas, while red ceiling light’s provide contrast for the animal’s body contour. The intensity of the signal depends on the area of the paw in contact with the platform and increases with the pressure applied by the paw. A high-speed color camera, positioned underneath the glass plate records the illuminated paw areas. The software then transforms each scene into a digital image. A multitude of static and dynamic parameters of gait can be computed which can be further clustered into the following four categories (30): I. General parameters of gait; II. Pain-related parameters of gait; III. Coordination-related parameters of gait; IV. Other parameters of gait. Table 1 provides an overview of the different gait parameters assessable via the CW. The table also contains a detailed description of the individual parameters’ meanings as well as the respective categories (I–IV) they can be assigned to.

**Table 1:** Gait parameters assessed via CatWalk in the rodent models of arthritic disorders included in this review.

Categories	Parameter	Explanation	References	Times assessed
I	Print Length (distance unit)	Length of the paw print	(32–39)	8/41 (20%)
I	Print Width (distance unit)	Width of the paw print	(32, 33, 35–38)	6/41 (15%)
I	Stride Length (distance unit)	Distance between two successive placements of the same paw	(32, 33, 35–38, 40–42)	9/41 (22%)
I	Base of Support (distance unit)	Distance between hindpaws or forepaws	(37)	1/41 (2%)
I	Print Area (distance unit^2^)	Area of the complete paw print	(32, 33, 35–38, 40, 42–53)	19/41 (46%)
I	Maximum Print (Contact) Area (distance unit^2^)	Maximum area of the paw in contact with the glass floor	(35–38, 50, 53–55)	8/41 (20%)
II	Total Paw Print Intensity (arbitrary units)	Total intensity of the paw print (Mean pixel intensity × number of pixels)	(56–62)	7/41(17%)
II	Swing Time (seconds)	Duration of the swing phase of the paw	(35, 36, 40, 45, 50, 51, 63–65)	9/41 (22%)
II	Stand Time (seconds)	Duration of the stand phase of the paw	(32, 33, 35, 36, 40, 42, 44, 45, 49, 51, 52, 66)	12/41 (29%)
II	Duty Cycle (%)	Percentage of the stand phase in a step cycle: [Stance time/(Stance time + Swing time)] x 100	(35–37, 40, 41, 43–46, 50, 52, 63, 64, 67, 68)	15/41 (37%)
II	Mean Intensity (arbitrary units)	Mean intensity of the paw print	(34, 42, 45, 69–72)^1^^,^^2^^,^^3^	7/41 (17%)
II	Maximum Intensity (arbitrary units)	Maximum intensity of the paw print	(38, 42, 44, 53)	4/41 (10%)
II	Mean Intensity at Maximum Contact (arbitrary units)	Mean intensity of the paw print at maximum contact of the paw	(33, 36, 40, 44, 48, 73, 74)^4^^,^^5^^,^^6^	7/41 (17%)
II	Maximum Intensity at Maximum Contact (arbitrary units)	Maximum intensity at maximum contact of the paw	(53, 66)	2/41 (5%)
II	Arthritic Paw Load (%)	Weight load (area x pressure) distribution between ipsilateral and contralateral paws	(75)	1/41 (2%)
II	Initial Contact Time (seconds)	Time from the start of the run to first contact of the paw with the glass plate	(35, 36)	2/41 (5%)
II	Maximal Contact Time (seconds)	Time from the start of the run to maximum contact of the paw with the glass plate	(35, 36)	2/41 (5%)
II	Anchor Paw Print Intensity ratio (%)	Calculated extent of anchor limb loading during walking	(65)	1/41 (2%)
II	Target Paw print Intensity ratio (%)	Calculated extent of target limb loading during walking	(65)	1/41 (2%)
II	(Dynamic) Weight bearing (arbitrary units)	Calculated force of a paw against the glass floor during walking, measured separately for all paws	(41, 52, 68)	3/41 (7%)
II	(Dynamic) Weight Bearing (%)	Percent weight bearing of one paw relative to all four paws	(41, 52, 68)	3/41 (7%)
II	(Dynamic) Guarding Index (%)	Difference in weight bearing (%) between the two hindpaws. Represents the shift in weight bearing between the two hindpaws.	(41, 52, 68)	3/41 (7%)
III	Normal Step Sequence Patterns (NSSP)	Specific sequences of paw placements during a step cycle	(38)	1/41 (2%)
III	Regularity Index (RI) (%)	Percentage of NSSP in the total number of paw placements (PP)	(37, 38, 40, 41, 46, 47, 49, 68)	8/41 (20%)
III	Phase Lags / Phase Dispersions (%)	Temporal relationship between footfalls of two different paws within a step cycle. It relies the initial contact of one paw (the Target) to the stride cycle of another paw (the Anchor).	(37, 38, 52)	3/41 (7%)
III	Phase Lag Variability (%)	Measure of the accuracy in the interlimb coordination	(37)	1/41 (2%)
IV	Step Cycle (seconds)	Duration of Stand Time + Swing Time of a paw	(37)	1/41 (2%)
IV	Print Positions/ Relative Paw Placements (distance unit)	Distance between the position of the hindpaw and the previously placed forepaw on the same side	(37, 38)	2/41 (5%)
IV	Single Stance (seconds)	Duration of contact with the glass plate with only one paw	(51, 52)	2/41 (5%)
IV	Swing Speed (centimeter /s)	Speed of the paw during the swing phase	(32, 33, 35–38, 40, 41, 43–45, 52, 54, 55, 63, 64)	16/41 (39%)
IV	Run Duration (s)	Total duration of the walkway crossing	(32, 33, 42)	3/41 (7%)
IV	Walking Speed/ Average Speed (centimeter/second)	Average speed during walkway crossing	(32, 33, 41, 45, 68)	5/41 (12%)
IV	Stand Index (%)	Speed at which the paw loses contact with the floor	(35, 36)	2/41 (5%)
IV	Paw Angle* (degrees)	Angle of the paw long axis against the horizontal plane	(35)	1/41 (2%)
IV	Paw Angle Movement Vector (degrees)	Angle between paw long axis and walking direction	(36)	1/41 (2%)
IV	Limb Idleness Index (LII) (%)	Index for OA associated pain, product of anchor and target print as well as swing time ratio	(65, 76)	2/41 (5%)

I: General, II: Pain-related, III: Coordination-related, IV: Other.

Note that the table does not include all parameters assessable via CW, but all of these which have been assessed by the researchers whose studies are included in this work. We excluded two studies which only listed significantly altered CW parameters but did not report all gait parameters which were analyzed (69, 77). Percentages presented are rounded.

1 Fouasson-Chailloux et al. (45): described as Paw Print Intensity in the study, not further specified, therefore assumed to be Mean Intensity.

2 Zahoor et al. (36): described as intensity, not further specified, therefore assumed to be Mean Intensity.

3 Fang et al. (73): described as Paw Intensity, not further specified, calculated as Log (Paw Intensity/weight), assigned to Mean Intensity.

4 Zahoor et al. (35): described as Paw Print Intensity in the study, not further specified, therefore assumed to be Mean Intensity at Max Contact.

5 Caglar et al. (32): described as Maximum Contact Intensity (%), not further specified, therefore assigned to Mean Intensity at Max Contact.

6 Kara et al. (33): described as Maximum Contact Intensity (%), not further specified, therefore assigned to Mean Intensity at Max Contact.

*Paw Angle against the horizontal plane can be assumed as both Paw Angle Movement Vector or Paw Angle Body Axis, not further specified.

The CW allows for reproducible combined evaluation of sensory and motor deficits and recovery during voluntary unforced movement of rodents. The CW’s feasibility to assess functional recovery in preclinical models of peripheral nerve injuries, one of the most important aspects of translational research, has been addressed before. However, its use in the field of preclinical arthritic models has not been reviewed yet, despite the high need for standardized assessment of functional deficits and -recovery in this field. Therefore, the aim of this review is to provide an overview over the rodent models of arthritic disorders that have been evaluated by means of the CW gait analysis system. We will discuss the various gait parameters that have been evaluated in the respective arthritis models, aiming to identify outcome measures that will enable the translation of basic preclinical discoveries into novel clinical therapeutics. This review will also shine a light on the specific gait alterations and provide insights into their interpretation and meaning in the context of the analyzed arthritic disorders. Finally, our review will also explore and discuss the CW’s potential in future studies addressing arthritic disorders.

## 2. Methods

In a first step we formulated three specific research questions we aimed to address in this review:

1. Which models of arthritic disorders have been evaluated with the CW gait analysis system?
2. Which CW parameters have been assessed most frequently in the respective models?
3. What are the optimum parameters for monitoring arthritic disorders in preclinical animal models?

As a next step, we developed a systematic search strategy. Using the term “CatWalk” we performed a comprehensive literature search in the PubMed database and the Web of Science Core Collection. Jana Ritter, Johannes Heinzel and Cosima Prahm searched, selected and evaluated the literature.

To identify all studies eligible for inclusion we defined detailed inclusion and exclusion criteria (Table 2). We included all original articles which made use of the CW or CW XT to evaluate gait changes in rodents (rats and mice) with any type of arthritic disorder. The full text had to be available in English and CW gait analysis had to be conducted at least at two different time points, e.g., preoperatively/pre-injection and postoperatively/post-injection. We excluded all studies assessing gait alterations in other models than arthritic disorders, e.g., peripheral or central nervous system injuries or disease, vascular or metabolic disease or other musculoskeletal disease. We also excluded all studies measuring age-related changes of gait, performing locomotion analysis in healthy animals and studies using other animals than rodents such as monkeys and ferrets. Further, review articles, expert opinions and publications without available full-texts were excluded. If studies used a chemical agent to induce disease, injection had to provoke any kind of arthritic disorder, e.g., publications inducing inflammation through intraplanar injection of an agent were excluded (Table 3). We also screened the respective studies’ reference lists to identify additional publications of relevance.

**Table 2:** Inclusion and exclusion criteria defined for the study selection process in this narrative review.

Inclusion criteria
Original articles
Rodent models: Mice and rats
Gait analysis performed with the help of the CW or CW XT
Arthritic disorders as leading component: Studies which made use of the CW or CW XT to evaluate changes of gait in rats or mice of arthritic disorders
Full-text available in English
Data assessment performed at least at two different time points, i.e., preoperatively and postoperatively
Exclusion criteria
Studies evaluating changes of gait related to other causes than defined in the inclusion criteria, e.g., injuries of the peripheral or central nervous system or the vascular system, internal disease (Diabetes) or other musculoskeletal disease than arthritic disorders (e.g., bone cancer pain, fracture)
No gait analysis at all or gait analysis in healthy animals only
Non-rodent models: Studies focusing on animal models other than rodents, e.g., ferrets, monkeys, humans
Review articles, expert opinion, abstract only
Gait analysis was not performed with CW or CW XT: Studies using another gait analysis system than the CW system, e. g. the DigiGait or the TreadScan for evaluation of gait
Complex injury patterns
Intraplantar injection of chemical agents with no direct relation to the joint

**Table 3:** Studies excluded from the narrative review (*n* = 8) after full text screening with reasons for exclusion.

Reference	Reason for exclusion
Patrignani et al. (72)	Model of inflammation induced by subcutaneous injection into plantar surface, no joint relation
Pitzer et al. (78)	Model of inflammation induced by intraplantar injection, no joint relation
Chen et al. (79)	Peripheral nerve injury: Sciatic nerve injury (sciatic nerve resection and repair)
Shepherd & Mohapatra (80)	Model of inflammation induced by intraplantar injection, no joint relation
Garcia et al. (81)	No performance of gait analysis: Evaluation of spontaneous locomotor activity by means of Actimeter Test
Taniguchi et al. (82)	Full text not available in English
Garrick et al. (83)	Locomotion analysis in healthy rodents
Zhao et al. (84)	Model of inflammatory pain induced by intraplantar injection, no joint relation

As it would break the limits of this review to present the data of all included studies, we selected representative studies for every model of arthritic disorders for more detailed analysis and discussion. Criteria considered for selection comprised study methodology, assessed gait parameters, follow up, number of evaluation time points, comprehensible presentation and completeness of assessed data, rodent type, number of available studies for the specific model. However, relevant data of all included studies is displayed in Supplementary Table S1 (Osteoarthritis models), Supplementary Table S2 (Monoarthritis models) and Supplementary Table S3 (Polyarthritis models), respectively.

To show the course of selected CW parameters more clearly, we extracted and prepared data regarding three of the most commonly assessed parameters, i.e., Print Area, Duty Cycle and Swing Speed, in figures. As it was often only possible to extract this data from the respective figures in the original study, 5–10% of variation is included. For the same reason, standard deviation or other measures of the amount of variation were not extracted. Not all authors divided the data assessed for the injured limb by a healthy, uninjured control limb or even by the preoperative values for the respective paw or limb. Whenever possible we calculated such a ratio from the extracted data (30). Preferentially, e.g., in case of unilateral osteoarthritis, the contralateral limb was used. If the contralateral limb was affected, e.g., in case of bilateral OA, an uninjured front limb was used to calculate this ratio.

Our review followed Deumens et al. classification of CW parameters who distinguished between General, Pain-related, Coordination-related and Other parameters of gait (30, 85). Several studies evaluated the effect of therapeutic strategies, e.g., analgesics, on gait behavior. These treatments and their consequences are presented in short when necessary and only in regard to gait alterations. When a study focused on the analysis of therapeutic strategies, data of control or sham rats was considered for data extraction and analysis.

## 3. Results

**Figure 1:**
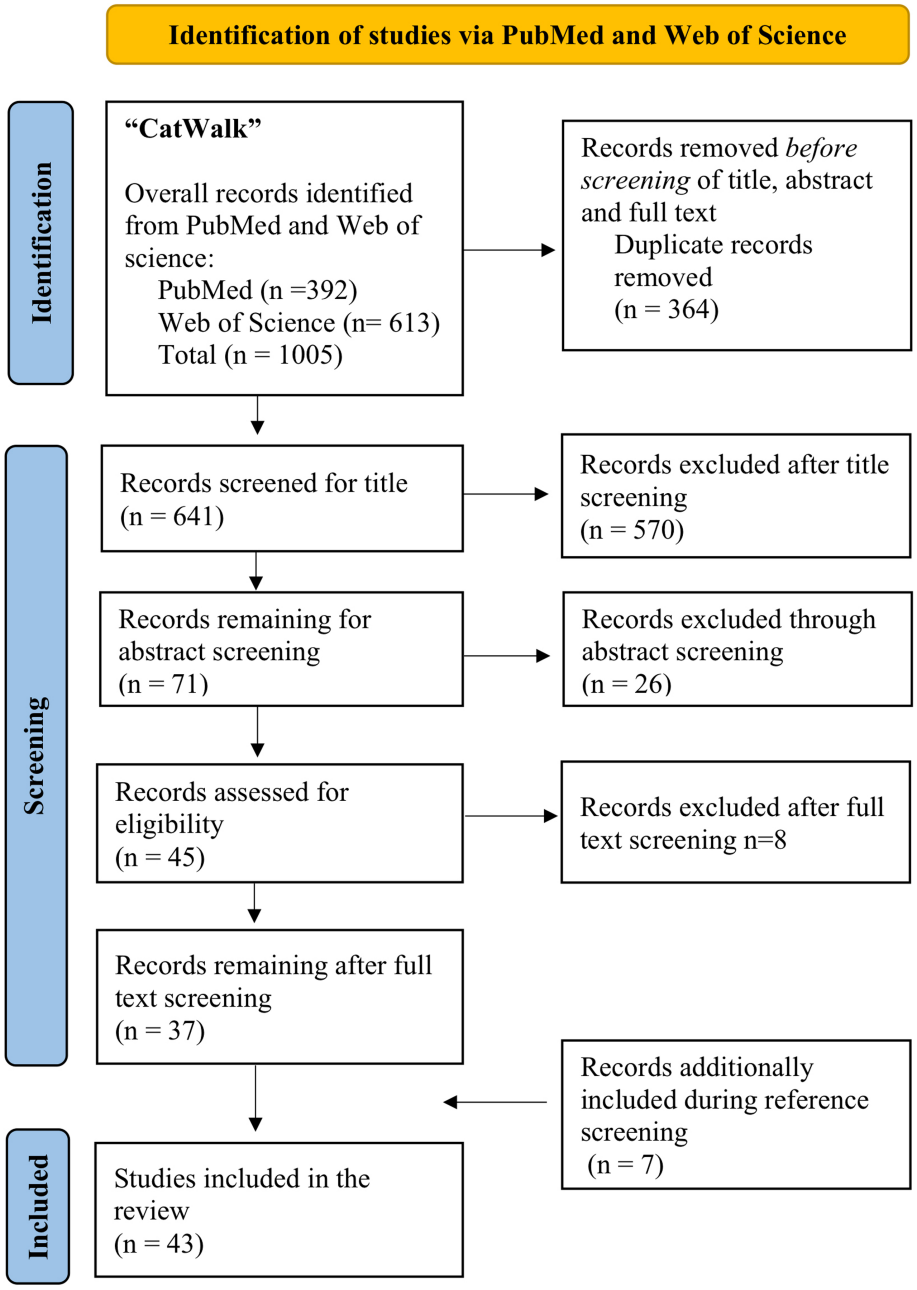
Flow diagram of the literature search according to the PRISMA guidelines (86).

Figure 1 displays the process of our literature research. Our search yielded 392 results in the PubMed database while 613 records were identified via Web of Science Core Collection, leading to a total of 1,005 results. After removal of duplicates 641 studies remained for further screening of title, abstract and full text. After application of inclusion and exclusion criteria, 37 records remained after full text screening for data analysis. Through review of references, 6 additional studies could be identified which met the inclusion criteria, leading to a total of 43 publications included in this review. The publication dates of the included studies range from 2007 to 2022.

**Figure 2:**
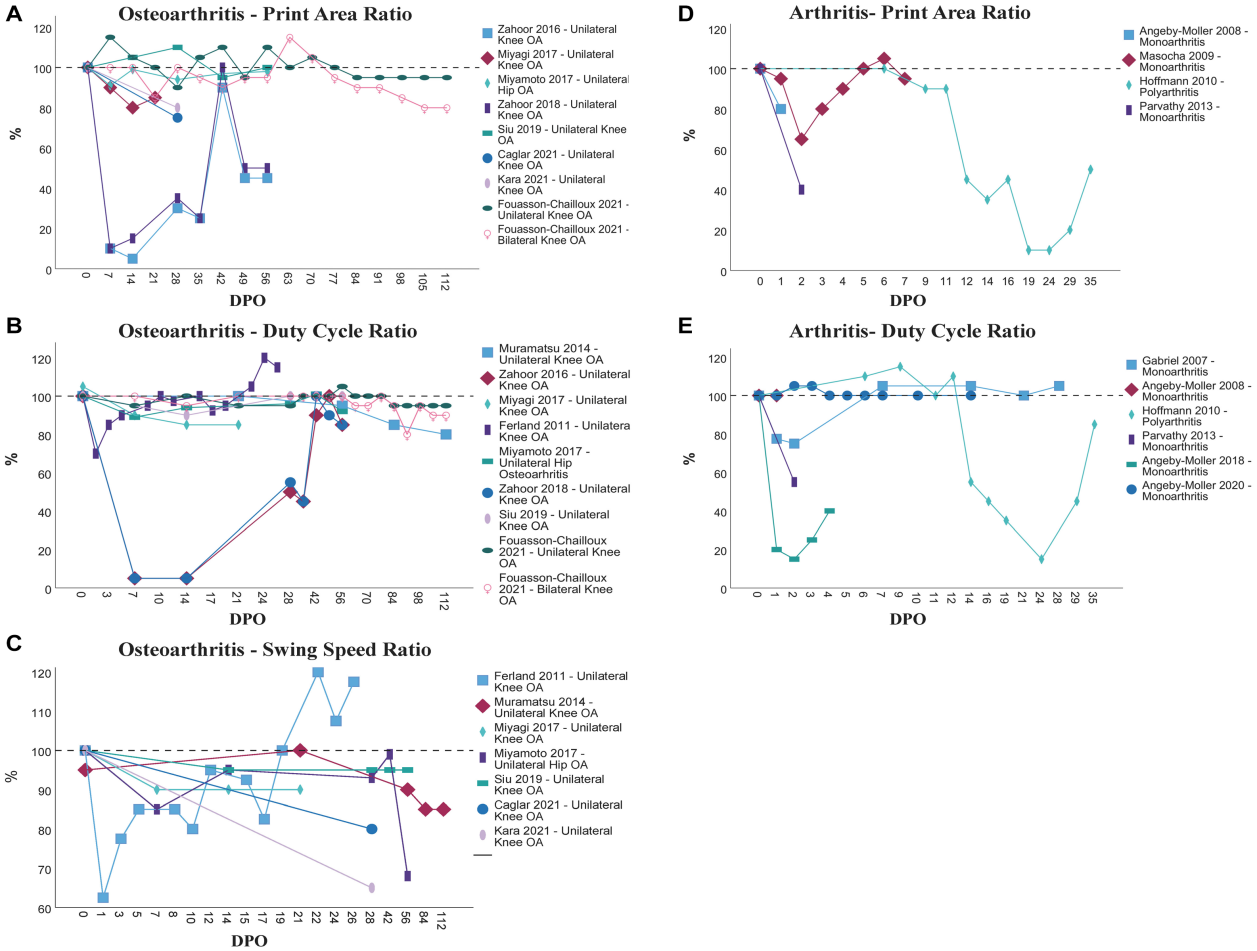
Comparison of the course of Print Area Ratio **(A + D)**, Duty Cycle Ratio **(B + E)** and Swing Speed Ratio **(C)** in different models of osteoarthritis **(A–C)** and arthritis **(D + E)** as assessed by the CW. OA, Osteoarthritis; DPO, Day(s) postoperative.

88% (36/41) of all papers included in this review assessed Pain-related gait parameters followed by General [25/41 (61%)], and Other parameters [21/41 (51%)] of gait (Table 4). Only 22% (9/41) of all analyzed studies measured Coordination-related parameters. Out of the 43 papers included in this review 40 (93%) also made use of other evaluation methods beside the CW. Figure 2 displays the course of Print Area Ratio (A + D), Duty Cycle Ratio (B + E) and Swing Speed Ratio (C) in different rat models of osteoarthritis and arthritis, respectively. We prepared no figure to display the course of Swing Speed Ratio in rodent models of arthritis since it would only have included one study.

**Table 4:** Frequency of gait parameters assessed in 41/43 studies included in this review.

Categories	Times assessed
I: General	25/41 (61%)
II: Pain-related	36/41 (88%)
III: Coordination-related	9/41 (22%)
IV: Others	21/41 (51%)

Parameters are sorted by category. This table excluded two studies which only listed significantly altered CW parameters but did not report the specific gait parameters that were assessed in total (69, 77).

### 3.1. Knee osteoarthritis

In rodent models of knee OA, the most common six assessed gait parameters were Swing Speed (12/31), Print Area (11/31), Duty Cycle (9/31), Stand Time (9/31), Swing Time (8/31) and % Total Paw Print Intensity (%TIPPI) (7/31).

In 2008, Ferreira-Gomes et al. investigated movement-induced nociception via the CW in a rat model of OA induced by the injection of Monoiodoacetate (MIA) into the left knee joint (56). They measured the %TIPPI of each paw for 31 days following OA induction. In osteoarthritic rats, the Total Intensity of the affected left hindlimb (LH) of the Total Intensity of both limbs remained significantly reduced throughout the whole study period compared to baseline, when weight load was equally distributed between both hindlimbs and compared to control groups. In summary, rats showed an extensive unwillingness to bear weight on the affected hindlimb. Moreover, OA rats exhibited a biphasic nociceptive response, with Total Ipsilateral Paw Print Intensity reaching a minimum at postoperative day (DPO) 3, increasing thereafter until the end of week 3, when again a significant decrease was observed (56). Correlation analysis between CW data and other behavioral test at DPO31, revealed a higher correlation of the Knee Bend Test, a modification of the Ankle Bend Test for the assessment of movement-evoked pain originating from the injured knee joint (87) with CW as between von Frey Testing and CW (56).

In 2012, Ferreira Gomes et al. (57) tried to verify if alterations in Paw Print Intensity as assessed with the CW are attributable to altered pain sensitivity after MIA injection into the left knee joint. Therefore, they determined the effects of Diclofenac, Lidocaine and Morphine on these gait changes in OA-induced rats at an early (Day 3) and late (Day 20) stage of OA evolution. All OA rats exhibited a significantly decreased Total Ipsilateral Paw Print Intensity before administration of the drugs at DPO3 and DPO20, respectively. CW data revealed that the analgesics Morphine, Lidocaine and Diclofenac alleviated changes of Ipsilateral Paw Print Total Intensity at different time points of OA evolution, indicating the existence of nociceptive behavior.

Adaes et al. published similar findings like Ferreira Gomes et al. (56, 57) but in a model of OA induced by two injections (Day 0 and Day 3) of collagenase into the left knee joint of Wistar rats (58). The %TIPPI was evaluated for 6 weeks. It should be noted, that in collagenase-injected rats maximal changes of %TIPPI occurred later and were less pronounced than those induced by MIA, but with the %TIPPI remaining reduced through the whole 6 weeks compared to the control group as well as experimental animals’ baseline values. Collagenase-injected rats showed a maximal decrement of %TIPPI at 1 Week Postoperative (WPO), increasing thereafter until WPO4, when %TIPPI decreased again until week 6. Correlation analysis between CW data and histological scoring revealed significant correlations between %TIPPI and different histological parameters at WPO1 and 6, showing more correlations at week 6. Consequently, nociceptive behaviors seem to be associated with OA like joint lesions.

In 2015, Adaes et al. (59) evaluated the %TIPPI via CW along with the expression of neuronal injury markers ATF-3, NPY and Neuropeptide SP over a period of 6 weeks. Changes in % TIPPI were consistent with previous findings (56–58). Repeated Gabapentin treatment starting at week 6 after OA induction (twice a day for 5 days, 30 mg/kg) significantly alleviated the nociceptive behavior and increased the % TIPPI. Moreover, the %TIPPI data significantly correlated with the increase of the neuronal injury marker NPY at WPO6 contrasting Ferreira Gomes’ results in 2012 (59, 60). It can be suggested that changes in %TIPPI observed at later stages of OA are predominantly related to neuropathic mechanisms of pain.

Ferland and her group investigated pain-related behavioral responses in male Sprague Dawley (SD) rats after either injection of MIA or surgically transection of the anterior cruciate ligament combined with a partial medial meniscectomy (ACLT + pMMX) of the left knee joints (63). Swing Time, Swing Speed and Duty Cycle were evaluated for both hindlimbs every 2 days for 4 weeks in both models of OA. MIA injected rats exhibited significant changes in all three CW parameters during the whole observation period. Swing Time of the ipsilateral limb was significantly increased while Duty Cycle and Swing Speed were significantly reduced. Moreover, rats guarded the osteoarthritic limb from mechanical stimulation by shifting weight load to the unaffected limb reflected in a shorter Swing Time, higher Swing Speed and increased Duty Cycle of the contralateral limb. However, while MIA-injected rats showed clear antalgic gait behavior, in the ACLT + pMMX model no significant differences between both hindlimbs could be observed within the 4 weeks, with the exception of Duty Cycle at DPO1.

In 2017, Miyagi et al. (43) evaluated the effects of a single i.p. administration of an anti- nerve growth factor antibody on gait changes of C57BL/6 mice in a MIA model of knee OA. Duty Cycle, Swing Speed and Print Area were assessed weekly for 5 weeks, starting at WPO3 after OA induction and compared between a group of MIA-injected mice treated with the abovementioned antibody, a non-treated group of MIA animals and a control group without OA. Consistent with previous findings in rats (56, 63), non-treated-osteoarthritic mice revealed significantly decreased ratios of ipsilateral and contralateral hind paw values not only of Duty Cycle and Swing Speed, but also of Print Area until WPO5, which were significantly different from control mice throughout the whole study.

In the past years, evaluation of General parameters of gait in MIA models mostly comprised assessment of Print Area and Max Contact Area. However, in 2021 Caglar et al. and Kara et al. extended the analysis of general gait parameters and measured Print Area along with Print Length, Print Width and Stride Length 4 weeks after the injection of MIA into the left knee joint of Wistar rats (32, 33). They further evaluated Mean Stand Time, Mac Contact Intensity, Run Duration, Average Speed and Swing Speed. In both studies injured left hindlimbs displayed significantly decreased Print Length, Print Width, Swing Speed and Average Speed compared to baseline. Caglar’s group also observed significantly decreased Print Area and increased Run Duration, while in Kara’s study Max Contact Intensity was significantly decreased. Furthermore, both groups reported insignificantly decreased Stride Length and increased Mean Stand Time. The gait behavior observed in this study confirms the findings of previous studies. Following MIA injection rats present reluctance to use the injured limb, which may be interpreted as adaptational mechanisms to movement-induced nociception. Of note, both studies featured small sample sizes and performed CW gait analysis only at WPO4 after MIA induction.

Siu et al. (44) used the ACLT model of OA to study the analgesic efficacy of topical applied Chinese herbal paste (DEAP) on knee pain over an 8-week treatment period. Stand Time, Print Area, Maximum Intensity, Swing Speed and Duty Cycle of the injured right hindlimb (RH) were examined biweekly in DEAP-treated rats, control rats with ACLT surgery but without DEAP treatment and sham rats. In contrast to Ferland’s findings (63) in Siu’s study control rats unveiled a significantly shorter Stand Time and a reduced Duty Cycle of the injured RH compared to non-osteoarthritic rats as early as 2 weeks after ACLT. Nevertheless, significantly reduced Print Area and Maximum Intensity of the injured limb of untreated OA rats became evident only at the end of the study at WPO8 compared to the sham group. Moreover, untreated rats exhibited significantly lower Swings Speeds of the injured limb starting from WPO6 compared with that of non-OA rats.

In 2020 Zhu et al. assessed changes of Stand Time and Max Contact Max Intensity for each hindlimb separately following ACLT of the right knee joint of C57/B6 mice for 6 months (66). As a consequence of OA induction mice avoided to bear weight on the injured limb reflected by a significantly reduced Stand Time and Max Contact Max Intensity compared to sham mice during the whole study period. During the onset of OA mice counterbalanced these gait disturbances and presented increased Stand Time and Max Contact Max Intensity on the contralateral limb until MPO1. However, this redistribution between the injured and non-injured limb was only observed at an early stage as the values of both hindlimbs gradually converged from MPO3 to MPO6.

Muramatsu et al. (77) performed the first detailed gait analysis via CW in a Destabilization of Medial Meniscus (DMM) mouse model of OA, measuring 21 different gait parameters of the hindlimbs at baseline and WPO, 3, 8, 12 and 16, in 2014. Until week 8 after DMM surgery on the left knee joint, C57BL/6 mice maintained normal gait behavior. Significant gait impairment was apparent from WPO12 until WPO16. DMM mice exhibited significantly reduced LH/RH ratios of Stand Time, Single Stance, Duty Cycle and Swing Speed, as well as increased Swing Time compared to baseline values and to sham operated mice. The overall analyzed gait parameters were not further specified, as authors only report gait parameters exhibiting significant alterations. However, this needs to be considered an important information in context of comparisons with other studies. We therefore recommend listing the specific analyzed gait parameters.

Malfait et al. (88) and Fang et al. (73) assessed Mean Intensity at Max Contact and Paw Print Intensity, i.e., presumably Mean Intensity of the hindlimbs following DMM surgery in mice for either 56 days or 10 weeks. In accordance with Muramatsu’s findings (77), Mean Intensity at Max Contact of DMM CD-1 mice remained constant up to DPO42, after which the CW system malfunctioned (88). Similarly, Fang’s group observed significantly lower Paw Print Intensity of the injured hindlimb of DMM C57BL/6 mice only at the end of the study at WPO10 compared to sham mice (73).

Fouasson-Chailloux and his group investigated gait impairment in unilateral and bilateral DMM-induced knee-OA in mice, in 2021 (45). Thus, they were the first providing gait data on surgical induced bilateral OA. Weekly, for 16 weeks following DMM surgery on the right knee joint (Uni DMM group) or both knee joints (Bi DMM group) Velocity (Mean Speeds), Print Area, Print Intensity, i.e., probably Mean Intensity, Stand Time, Duty Cycle, Swing Time, Swing Speed of all limbs were evaluated. In both groups, Velocity was significantly reduced at WPO1 compared to the control group, however, it recovered to baseline until WPO2. The Bi DMM group presented increased Duty Cycle of both hindlimbs at WPO1 compared to control mice, indicating increased time spent on the injured hindlimbs. In contrast, in the Uni DMM group, Duty Cycle was significantly increased on the contralateral LH and right forelimb (RF) at WPO1, recovering thereafter. Moreover, in the Uni DMM group Print Area of the contralateral LH was significantly increased and remained significantly different from the control group from WPO1 until WPO16 with the exception of WPO2-3. In contrast, In the Bi DMM group no significant alterations of Print Area of all limbs compared to control group were found. Authors did not observe any significant differences between control group and Uni or Bi DMM group for Stand Time, Swing Time and Swing speed for all limbs until WPO16. This contrasts the finding of Muramatsu’s group, who detected significantly reduced Stand Time and Swing Speed as well as increased Swing Time at WPO12 after unilateral DMM compared to sham operated mice (77). While rats with unilateral induced OA exhibited several gait disturbances mirroring redistribution of weight bearing towards the contralateral uninjured limb, rats with bilateral OA presented a more symmetrical gait which may be attributed to the inability of gait adaptation as both joints are injured.

In 2021, Westhof et al. (34) were the first analyzing gait in a rat DMM model of OA. After female SD rats underwent DMM surgery on their right knee joints, Print Length was analyzed at DPO6, 34, 62 and 90. The RH/LH ratio of Print Length was significantly decreased at DPO6 compared to control group, recovering until DPO34. Subsequently, significant differences between the Print Length of DMM injured rats and control rats were absent at the following evaluation timepoints until the end of the observation period. These findings are in a line with data reported by Fouasson-Chailloux et al. (45) in the DMM mouse model of OA, where first gait disturbances revealed at WPO1 after DMM surgery. Of note, Fouasson-Chailloux et al. and Westhof et al. were the first detecting gait alterations early after DMM induction, contrasting findings of Muramatsu’s, Fang’s and Malfait’s groups which observed first gait disturbances at later timepoints only.

In 2016, Zahoor et al. (35) developed a surgically induced intraarticular fracture model mimicking post-traumatic OA and investigated the consequences on gait behavior. Left knee joints of eight male SD rats underwent intraarticular fracture of the medial tibial plateau induced with the help of a surgical scalpel and a hammer. The fracture was immediately internally fixated with two needles to resemble clinical treatment of the fracture. General parameters Print Length, Print Width, Max Print Area, Mean Print Area, Stride Length, pain-related parameters. “Paw Print Intensity” i.e. presumably Mean Intensity, Stand Time, Duty Cycle, Swing Time, Initial Contact Time, Max Contact Time and other parameters Swing Speed, Paw Angle and Stand Index of the injured limbs were assessed for 8 weeks post-surgery. Authors observed significant alterations of nearly all gait parameters besides Print Width, Initial Contact Time, Paw Angle and Swing Seed following the induction of intraarticular fracture. Overall, rats exhibited significant gait impairment from WPO1-5, with most pronounced changes from baseline unveiling in the first 2 weeks. Almost all parameters recovered to levels near baseline until WPO4-8 with the exception of Print Length and Max and Mean Print Area. In the first 2 weeks especially, Stride Length and Paw Print Intensity were significantly reduced while Max Contact Time and Stand Time were significantly increased, recovering thereafter, almost reaching preoperative levels at WPO4-6. Duty Cycle, Max and Mean Print Area, were significantly decreased, while Swing Time and Stand Index were significantly increased from week 1–5, recovering until WPO 6–8. Although Max and Mean Print Area recovered, they were still reduced at WPO8 compared to baseline but without reaching statistical significance. Interestingly, Print Length was the only gait parameters which was significantly reduced at WPO7-WPO8, Gait disturbances reflect joint disfunction and the rats attempt to reduce weight bearing on the operated limb, while simultaneously maintaining balance and stability as a consequence of fracture induction. Correlation analysis between gait data and the histological Mankin Score of the injured limbs performed at WPO8 failed to reveal any correlations between Print Length and histological evident articular cartilage destruction around the consolidated fracture side. Of note, Histology was evaluated at WPO8 only and sample size was quite small comprising only 8 SD rats. Moreover, there was no control group, therefore the effect of the surgery itself on gait remains unclear.

In 2017, Zahoor et al. evaluated the effect of low-intensity pulsed ultrasound (LIPUS) on the same gait parameters compared to non-treated control SD rats in the intraarticular fracture model of OA (36). In a line with their previous findings maximal change from baseline was observed in the first 2 weeks after OA induction of non-treated control rats. Max Print Area, Print Area, Duty Cycle, Paw Angle with walking direction and Stride Length were drastically reduced. However, Paw Angle and Stride Length exceeded normal levels from WPO4-8 and WPO 6–8. In contrast to previous findings, most gait parameters of the injured limbs of control rats did not reach baseline values nor exhibited marked recovery until the end of the observation period at WPO8. Rats treated with LIPUS revealed less pronounced and fast recovery of gait impairment with most pronounced improvements compared to non-treated rats at WPO7-8.

### 3.2. Hip osteoarthritis

While all previous described publications analyzed gait in rodent models of knee OA, Miyamoto et al. (69) were the first group using the CW system to investigate pain-induced gait changes in a model of Hip-OA, in 2017. Correspondingly, they examined 21 gait parameters following the injection of 2 mg of MIA into the right hip joints of Sprague Dawley rats for 56 days. In the MIA-group the RH/LH ratios of Stand Time, Duty Cycle, Swing Speed, Print Area, Max Contact Area and Max Intensity decreased significantly as early as DPO7 and remained reduced for the rest of the observation period compared to those of the sham-operated rats, although they increased steadily until DPO56. These gait changes may be an indicator of movement induced nociception, but also of altered mechanics of the hip joint triggered by OA.

### 3.3. Monoarthritis

In rodent models of MA, Regularity Index (6/9), Duty Cycle (6/9) and Print Area (5/9) were the most frequently evaluated gait parameters. According to our review, the CW system has been used to evaluate gait behavior in rodent models of knee and ankle joint MA. The average follow-up in MA models was 9 days.

In 2007, Gabriel et al. (37) determined gait abnormalities in a rat model of Carrageenan (CARR) induced acute inflammatory knee pain over 48 h. Mean Intensity, Duty Cycle, Relative Paw Placements as well as the Coordination-related parameter Phase Lag exhibited significant changes from preoperative values at 24 and 48 h after CARR injection into the right knee joint. While Mean Intensity of the affected limb was significantly decreased, it remained unchanged on the contralateral limb compared to baseline. The Duty Cycle of the injured limb was insignificantly reduced, whereas it was significantly elevated on the non-injured limb compared to baseline. CARR injection did not alter the Base of Support (BoS) of the hindlimbs. Relative Paw Placement of the injected limb was significantly increased compared to baseline. This implicated that the rats were unable to maintain their natural preference of superimposed placement of both ipsilateral paws to maintain secure footing during ambulation (70). Analysis of interlimb coordination revealed significantly increased Hind-Girdle Phase Dispersions (PD) (RH affected vs. LH non-affected) at 24 and 48 h as compared to baseline, representing a delayed initial contact of the affected RH in the step cycle of the contralateral LH. Phase Lag Variability and Regularity Index (RI) remained unchanged. Moreover Gabriel et al. observed significant changes of Swing Speed, Max Print Area, Print Area, Print Width, Print Length, however this data is not shown. Correlation analysis between CW data and Von Frey data of the affected RH, revealed strongest correlation between Von Frey data and Ipsilateral Mean Intensity, followed by Print Width, Print Length, Print Area, Duty Cycle, Swing Speed and Max Print Area (37).

Gabriel and her group extended their previous analysis of pain-related gait changes in a rat model of acute inflammatory pain to an evaluation in a model of chronic inflammatory knee joint pain, in 2009 (67). Similarly, CARR was injected into the right knee joint of rats. Mean Intensity and Duty Cycle of the RH were evaluated for 4 weeks. In contrast to previous finding in a model of acute joint pain (37), Duty Cycle and Mean Intensity were significantly decreased compared to pre-operative values at DPO1 only, recovering to baseline values thereafter. There were no significant correlations between CW data and Von Frey data.

Angeby-Moller et al. (46) were the first to use the CW system in a rat model of ankle-joint Monoarthritis (MA) induced by CARR injection into the left tibiotarsal joint. Print Area, Mean Intensity, Duty Cycle of all four limbs as well as RI were examined for 24 h after injection. Monoarthritic rats revealed significantly decreased Print Area, Mean Intensity and Duty Cycle of the affected LH compared to baseline and compared to the contralateral limb, whereas remarkable changes in the other limbs were absent. Authors observed maximal change of CW parameters from baseline at 5 h post-injection, increasing thenceforth. In summary, monoarthritic rats tried to reduce standing time and weight loading of the affected limb. RI significantly decreased by 70% until 5 h indicating disturbances of interlimb coordination during ambulation, which is surprising as maintenance of coordination is inherent to central nervous system (71). Interestingly, all gait parameters did not recover to baseline until the end of the observation period. Rofecoxib and Morphine showed a dose- and time dependent effect on gait disparity and could alleviate all CARR-induced gait changes (46).

Masocha and Parvathy published the first report on weight bearing changes in a mice model of Lipopolysaccharide (LPS) induced ankle joint MA, in 2009 (47). They evaluated Mean Intensity at Max Contact and Print Area of all four limbs as well as RI for 7 days following the injection of LPS into the right ankle joint of C57BL/6 mice. In contrast to Angeby-Moller (46), who found changes in gait parameters of the affected limb only, Masocha et al. observed significant redistribution of weight loading from the affected RH towards the contralateral hindlimb and the forepaws. While Mean Intensity at Max Contact and Print Area of the affected RH decreased following LPS injection, reaching a minimum at DPO2, they increased on the unaffected LH and the forepaws compared to preoperative values. Nevertheless, significant gait alterations were evident only in the first 3 days after MA induction. All parameters recovered back to baseline until DPO7. In accordance with Gabriel et al. (37), the RI remained unchanged. Intraperitoneal Indomethacin treatment alleviated gait disturbances after MA induction.

In 2013, Parvathy and Masocha, performed a comprehensive analysis of static and dynamic gait parameters of female C57BL/6 mice with Complete Freund’s adjuvant (CFA) induced MA of the right ankle joint for 7 days (40). CFA-injected mice exhibited significantly reduced Print Area, Mean Intensity at Max Contact as well as Stand Time, Duty Cycle and Swing Speed ratios [RH (arthritic)/LH (non-arthritic)] compared to baseline, reaching lowest values at DPO3-4. In contrast, Swing Time ratio was significantly increased. All gait alterations subsequently recovered to baseline until the end of the observation period. These findings are in a line with what has been previously observed in the LPS and CARR-induced models of MA (37, 46–48). Stride Length was not significantly changed from baseline until DPO7. Stride Length is the product of Swing Time and Swing Speed. As Swing Time significantly increased and Swing Speed decreased following CFA injection, their product (Stride Length) remained constant. Maintaining constant Stride Length may reflect the rats attempt to preserve balance and secure foot placements during locomotion when limb function is impaired. Moreover, rodents tend to place their hind paws at the same position previously vacated by the front paws when moving forward, enabling save footing (74). In addition, the authors also observed a significantly reduced RI at DPO2 as some rats completely avoided using the injured limb during ambulation. Therefore, we assume, that the reduced RI is attributed to the complete loss of usability of the affected limb rather than a coordination disorder. Treatment with Indomethacin i.p. relieved CFA-induced static and dynamic gait changes in a time-dependent manner (40).

In 2018, Angeby-Moller et al. (68) evaluated weight loading of the paws during movement, interlimb coordination as well as Walking Speed and Duty Cycle for 4 days post CFA-injection into the left tibiotarsal joint of Wistar rats. To determine weight loading of the distinct paws, authors multiplied the Max Print Area with the Mean Intensity of each paw. Results were presented as the Percent Dynamic Weight Bearing of one paw relative to all four paws. Dynamic Guarding Index (%) displayed the difference in percent Dynamic Weight Bearing between the non-injected right paw and the CFA-injected left paw. CFA injection resulted in significant alterations of all gait parameters during the whole observation period, unveiling maximal change from baseline at DPO 1–2, slightly reverting thereafter. The RI was reduced to 35% at DPO2 as some rats refused to load the affected limb. Walking Speed of CFA-induced rats was significantly decreased compared to control rats. While Duty Cycle of the affected LH was significantly reduced, Duty Cycle of the contralateral paw as well as of both forepaws were significantly increased. Complementing these changes, monoarthritic rats also significantly reduced Dynamic Weight Loading on the injured limb while increasing loading on contralateral limb and the forepaws during ambulation when compared to control rats, representing antalgic gait adaptation. Orally administered Naproxen significantly alleviated CFA-induced changes of the Guarding Index in a dose-dependent manner, whereas Pregabalin failed to do so over the entire study period.

In 2020, Angeby-Moller et al. (41) studied pain-induced gait changes in C57BL/6 mice for 14 days after injection of CFA into the left ankle joint. In monoarthritic mice Dynamic Weight Bearing and Duty Cycle of the affected LH were significantly reduced, while they were increased on the contralateral non-injured RH and RF compared to control mice. Interestingly, in contrast to previous reports, mice did not exhibit increased Dynamic Weight bearing and Duty Cycle of the left forelimb (LF) (68). Only 3 legs were involved in gait compensation. Furthermore, the Guarding Index (non-affected RH - affected LH) of CFA-injected mice was significantly increased throughout the whole observation period, as previously reported for rats (68). The RI of CFA-injected mice was significantly reduced at all time points post-injection, showing a maximal reduction of 90% at DPO1. In contrast to previous findings in rats, monoarthritic mice maintained constant Walking Speed as well as Stride Length for 14 days (68).

Gait analysis via CW was further performed in monoarthritic rats with different types of hypertension to analyze the impact of hypertension on pain modulation und sensitivity (75). After different models of hypertension were established in different rat strains, MA was induced through injection of CFA into the left tibiotarsal joint. 10 days after MA induction, the percentage of arthritic hindpaw load of Spontaneously Hypertensive Rats (SHR), Angiotensin II Induced Hypertensive Rats (ANG) and normotensive control rats was assessed to determine movement-induced nociception. Normotensive as well as hypertensive rats displayed altered gait with reduced weight loading on the injured hindlimb following MA induction. However, in SHR rats, loading on the arthritic hind paw was less reduced compared with their normotensive controls, whereas in ANG rats significant differences of arthritic hind paw load percentage compared with the normotensive controls were absent.

### 3.4. Polyarthritis

In contrast to models of Osteo- and Monoarthritis, which have been widely studied via automated gait analysis, we identified only 3 studies (33, 37, 87) which made use of the CW system to evaluate functional deficits in rodent models of polyarthritic disease such as RA.

In 2010, Hoffman et al. evaluated Print Area, Duty Cycle and RI in a model of Pristane-induced Polyarthritis (PIA) for 35 days (49). Intradermal Pristane injection at the base of the tail of Dark Agouti rats induced Arthritis in the RF as well as both hindlimbs at DPO11-12 reflected by an increasing Arthritis Score, based on macroscopic swelling and erythema of peripheral joints, as previously described (89). The LF remained unaffected. First gait abnormalities became apparent 1–3 days before the Arthritis Score increased. Accordingly, Pristane injection resulted in a remarkable reduction of the Print Areas of both hindlimbs at DPO9 and of the RF at DPO11. Additionally, a decrease of the LH and RF Duty Cycles was observable. When compared with saline-injected control rats, Print Areas and Duty Cycles of the affected limbs of Pristane-injected rats were significantly reduced from DPO12 until DPO35. However, after reaching minimum values at DPO24, they gradually increased until DPO35. Simultaneously, the unaffected limb of Pristane-injected rats exhibited a larger Print Area and higher Duty Cycle compared to that of control rats, suggesting a compensatory behavioral response. In regard to interlimb coordination, authors observed a significantly decreased RI of Pristane-injected rats from DPO12 until DPO35, reaching a minimum of 40% at DPO24 and increasing thereafter. Correlation analysis between semi-quantitative Arthritis Score and CW data confirmed significant correlations between Arthritis Score and Print Area as well as Duty Cycle. Systematic kinetic analysis between gait changes, histological findings and clinical variables confirmed that gait changes followed the clinical course of PIA. Although no other external signs of Arthritis could be detected when first gait changes occurred, histological signs of tenosynovitis in the joint were already evident.

Hayer et al. (38) performed gait analysis to investigate functional deficits in a mouse model of RA, in 2016. Static, dynamic and Coordination-related parameters of gait of female human TNF alpha transgenic mice (hTNFtg mice) were assessed from week 5 to week 15 after birth. Gait disturbances were apparent as early as 5 weeks after birth at an early inflammatory stage of Arthritis. Print Length, Print Width, Print Area and Maximum Intensity of front and hind paws of hTNFtg mice were significantly reduced compared to Wildtype (WT) mice. In regard to coordinated-related parameters, the authors observed significantly increased Phase Dispersions of diagonal pairs in hTNFtg mice suggesting that the hind paws had delayed contact with the floor. For NSSP, Print Positions and the RI significant differences compared to WT mice could not be observed. As Arthritis progressed in hTNFtg mice, aggravation of functional deficits was evidenced by a further reduction of Print Width and Length and further changes in interlimb coordination between week 6 and 15 after birth. Print Positions of right and left paws significantly increased indicating that mice placed their hind paws increasingly further behind the position previously vacated by the front paws. Although the RI exhibited a slight decrease and Phase Dispersions of diagonal pairs gradually increased from week 6 until week 15 after birth, this was not statistically significant when compared to wild-type mice. Linear regression analysis between Print Width and Length and distinct histopathological processes such as synovial inflammation, bone erosion, proteoglycan loss of the articular cartilage in the tarsal joints of the hindlimbs of 6, 10, 15 weeks old hTNFtg mice reveled significant associations between Print Length, Print Width and bone erosion as well as cartilage damage. However, statistically significant associations between Print Width and Print Length and synovial inflammation of the hind paws were not observed. A 5-week treatment of 10 weeks old hTNFtg mice with the anti-TNF antibody Infliximab i.p. ameliorated gait changes and reduced functional deficits until week 15 after birth (38).

In 2019, Mausset-Bonnefont et al. (42) used the CW to evaluate locomotion deficits in the well-established Collagen-induced model of RA. Accordingly, Collagen Type II was injected at the base of the tail of DBA/1OlaHsd male mice and Print Area, Stride Length, Max Intensity, and Stand Time of the hind and front paws were assessed for 49 days post-induction. Of note, authors display gait alterations of the hind paws only, as the front paws were similarly affected. In a line with Hayer’s findings Print Area of the paws of Collagen-induced arthritic mice gradually decreased from DPO28 until DPO49, compared to naïve mice. According to the authors, Stride Length, Stand Time and Max Intensity were also significantly decreased while Run Duration remained unchanged, however this data is not shown. Mausset-Bonnefont et al. were the first group performing correlation analysis between gait data and Clinical Arthritis Score based on peripheral joint swelling and redness (90, 91) in the Collagen-induced model of RA. Significant inverse correlation between Print Area and the clinical score of Arthritis was observed. The reduction of Print Area paralleled with a significant increase of Clinical Arthritis Score from DPO28 until DPO49. These findings are comparable with what has been previously reported for the PIA model (49). Methotrexate Treatment was able to alleviate collagen-induced gait changes. Noteworthy, as histological examination focused on ankle joints and behavioral testing mainly involved measurements of the hindlimbs, it remains unclear, how forelimbs were affected by arthritis.

## 4. Discussion

The fully automated CW gait analysis system, originally applied to study functional recovery in preclinical models of central nervous injury has gained growing interest in the scientific community (29, 92). Over the last decades, gait analysis became an established behavioral endpoint in preclinical research not only in the evaluation of animal models of central or peripheral nervous injury but also of musculoskeletal disorders. In this review we summarized the broad variety of different rodent models of arthritic disorders which have been evaluated using the CW system. According to our results, by now, rodent models of Mono, Osteo- and Rheumatoid Arthritis have been evaluated by means of the CW gait analysis system. In these models, the application of the CW ranges from the measurement of pain-related behavior and functional deficits arising from arthritic disorders to the evaluation of new therapeutic strategies. Pain is the first and major symptom of patients with arthritis, severely affecting the daily life and the main reason for treatment initiation (5–7, 10, 93). Nevertheless, adequate therapies are still lacking. This reflects the need to evaluate behavioral outcomes in preclinical models that parallel with the symptoms observed in OA patients to enable better clinical translation and clinically relevant evaluation of treatments.

### 4.1. Gait analysis in rodent models of unilateral limb injury

#### 4.1.1. General parameters of gait

Rodents with chemically or surgical induced knee OA displayed significant reductions of the General parameters Print Area, Print Length, Print Width, Max Contact Area and Stride Length (32–36, 43, 44, 50, 51, 54, 55). Overall, alterations of these General parameters are concordant with the presence of antalgic gait. Clinical examination of gait in knee OA patients also revealed decreased Stride Length (94, 95). Different drugs such as Morphine, Tramadol, Anti-Nerve Growth Factor antibody or Sigma Receptor antagonists, know to effect peripheral and central pain mechanisms (96–99), as well as i.a. injections of Astaxanthin, HA and Corticosteroids at the knee were able to alleviate the observed alterations of General gait parameters (32, 43, 50, 54, 55). This indicates that nociception may also have an impact on General parameters of gait such as Print Area, Print Length, Print Width, Max Contact Area and Stride Length indicating their usefulness and relevance in the evaluation of pain-related behavior and antinociceptive treatments.

#### 4.1.2. Pain-related parameters of gait

Analysis of Total Paw Print Intensity expressed as %TIPPI revealed a significant decrement after chemical induction of unilateral OA using MIA or Collagenase, which outlasted the whole observation time (56–60). The TIPPI changes with contact area and pressure applied by each paw, thus reflecting weight loading of the limbs. Reduction of %TIPPI, may therefore indicate guarding behavior of the injured limb as pain may be triggered through the mechanical stimulation of the joint during the walking process. Weight is redistributed between the injured and non-injured contralateral limb in order to protect the injured limb from painful loading. These gait modifications are consistent with findings in humans with unilateral knee OA (100, 101). Weight loading has been previously stated as an indirect measure of pain in rodent models of Arthritis (24, 102–110). Although, gait changes may be a correlate of joint pain, gait abnormalities may also be attributed to altered joint mechanics, joint dysfunction and joint stiffness due to progressive cartilage and bone destruction (22). Correlation analysis between the CW and the Knee Bend Test, used to assess movement-evoked pain originating from the injured knee joint (87) and the Von Frey Test, revealed higher correlation between CW data and Knee Bend Test data as with the Von Frey Test at DPO31 (56). These results indicate that the reduced %TIPPI of the injured limb is associated with pain arising directly from the knee joint. Interestingly, changes in %TIPPI followed a biphasic pattern reaching minimums at DPO3 and at the start of WPO4 in the MIA model of OA. In a collagenase model of OA, biphasic alterations in %TIPPI were comparable, however, they occurred later and were less pronounced (58, 59). These findings are consistent with the biphasic response reported for chemically induced models of OA. Accordingly, the nociceptive response is subdivided into an acute early inflammatory phase and a chronic phase characterized by progressive structural joint destruction and a neuropathic pain component (102, 103, 106, 108, 109, 111–115). For OA patients mixed pain states with both inflammatory and neuropathic components of pain are widely reported (116, 117). Changes of %TIPPI were able to capture this response pattern. Morphine (s.c.) and Diclofenac (p.o) significantly alleviated %TIPPI of arthritic rats at different stages of disease, indicating overall pain perception in the MIA model of OA as well as distinct mechanisms underlying pain. Analogous, in an collagenase model, Diclofenac (p.o) significantly improved the %TIPPI at disease onset, when synovial inflammation processes were predominant (58). It failed to be effective as OA progressed and clear histopathological OA lesions were apparent. Consequently, it can be hypothesized that first changes of %TIPPI are influenced by inflammatory induced-nociceptive pain sensation. As primary afferent nerve fibers innervate the knee joint, damage of joint structures may activate articular nociceptors, eliciting pain. Various mechanisms including the release of inflammatory mediators in response to damage of joint components were proposed to cause altered sensitivity of articular nociceptors in OA. As a consequence, mechanical threshold for activation of nociceptors is reduced resulting in enhanced pain sensitivity upon application of mechanical stimulation (116, 118, 119). It was further shown that injection of MIA itself induces sensitization of nociceptors (120). Association of inflammatory processes such as synovitis and effusion and pain sensitization were also observed in clinical research of knee OA patients (121). In contrast, the anti-convulsant Gabapentin, proven to be effective in the therapy of neuropathic pain (122, 123), was able to ameliorate % TIPPI at 6 weeks post-OA induction when extensive articular cartilage degeneration and areas of exposed subchondral bone were apparent (59). In addition, CW data significantly correlated with the neuronal injury marker Neuropeptide Y (NPY) at this timepoint. Moreover, when investigating the influence of glial activation in OA models, a process contributing to neuropathic pain (124–127), administration of Fluorocitrate at WPO6, an inhibitor of glial cell activation, was also able to attenuate alterations of %TIPPI (61). Consequently, these observations suggest the direct damage of neurons innervating the joint and subsequently the contribution of neuropathic pain mechanisms on gait changes as disease progresses. In addition, significant associations between %TIPPI and histology has been reported at 6 weeks after injury (58), evidencing that gait disturbances also parallel with histological changes at this time point. Therefore, at advanced stages of OA gait changes might be influenced by neuropathic pain mechanisms as well as by altered joint mechanics due to clear joint destruction, which might in turn impact or even reinforce pain perception.

According to the studies included in this review, the course of Stand Time, Duty Cycle and Swing Time, also underlined a clear limping behavior of rodents with unilateral knee OA, maintained throughout the respective observation periods. Gait alterations were comparable among the studies investigating knee OA. Of note, in the MIA models, gait parameters showed a trend towards the previous reported biphasic response pattern. Following the induction of knee OA in rats, Stand Time and Duty Cycle of the injured limb decreased while Swing Time increased (43, 44, 64, 66, 77, 102). In contrast, on the non-injured contralateral limb opposite effects have been reported, attributed to compensatory gait alterations (63, 64, 66). Due to antalgic gait, also Swing Speed of the injured limb decreased (43, 44, 63, 64, 77) while it increased on the contralateral limb (63, 64). Oral Celecoxib treatment improved Duty Cycle and Swing Speed at the acute inflammatory phase of OA, but lacked effects at later timepoints, indicating the presence of acute nociceptive pain at this timepoint. Interestingly, Tschon’s group reported a significant amelioration of Stand Time, Swing Time, Print Area and Single Stance following the intraarticular injection of the corticosteroid Triamcinolone acetonide (TA) and a combination of Hyaluronic acid-chitlac matrix enriched with TA. Subsequently, these findings indicate the influence of pain origination from the knee joint on these Pain-related gait parameters. It can be suggested that rats suffering from knee OA, modify their gait to protect their injured limb from mechanical stimulation in order to minimize pain sensation. Overall, these findings of altered mechanical loading are consistent with gait characteristics of humans suffering from unilateral knee lesions (26, 128, 129).

When Zhu et al. (66) evaluated the Pain-related parameters Stand Time and Max Contact Max Intensity of both hindlimbs in the ACLT mice model of OA, they observed clear gait compensation between the hindlimbs only at MPO1. Both parameters were decreased in the injured limb and increased on the contralateral limb. However, at later timepoints they also observed a decrease of Stand Time and Max Contact Area of the non-injured limb. This may indicate an influence of altered mechanical loading on OA progression. In addition, as disease progressed, the decrease of gait parameters on the contralateral limb paralleled with histological evidence of bone thickening in the non-injured limb. Antalgic gait behavior may serve rats to reduce pain perception, nevertheless, in the long term, gait modification may lead to an increase in pain, as altered mechanical loading may injure contralateral knees and adjacent joints. CW gait analysis may help elucidate early OA-related changes in the contralateral unaffected limb. Analogously, unilateral knee OA has been previously reported as a risk factor for the development of bilateral OA in humans. However, the influence of compensatory gait and altered mechanical joint loading on contralateral knees in patients with unilateral knee OA is still unclear (130–134). Further gait analyzes in preclinical rodent models of OA may provide new insights on the impact of altered mechanical loading due to unilateral knee OA as disease progresses and may help in the development of preventive treatments. Of note, changes in the contralateral limbs also need to be considered when using the contralateral limb as internal control, as previously stated by other authors (134).

Comparable with findings in models of OA, pain behavior of monoarthritic rodents is evaluable via the General parameters (I), Pain-related (II) and Other parameters of gait (IV). Unilateral acute joint inflammation leads to redistribution of weight load towards the non-injured limbs. Overall, monoarthritic rats present reduced Duty Cycle and Stand Time as well as prolonged Swing Time and decreased Swing Speed of the injured paw (32, 35, 36, 42, 43, 56, 69, 97). Rodents limit paw contact time while at the same time reducing overall weight load of the arthritic leg as Print Area and Mean Intensity drop. Subsequently, they tend to favor the non-injured limbs, leading to limping gait presenting with prolonged Stand Time and Duty Cycle and shortened Swing Time of the healthy legs. These temporal alterations parallel with greater Print Area and higher Paw Print Intensity of the healthy limbs. Interestingly, while rats mostly involve all limbs in gait compensation, in mice with CFA induced ankle joint arthritis, the front paw of the non-injured side tends to be unaffected (32). This might indicate different compensational behavior between mice and rats.

Studies revealed that gait alterations monitored by the Pain-related CW parameters following the induction of MA can be significantly attenuated by pain medication such as Indomethacin (43). In a line, CW data demonstrated significant correlations with Von Frey Test data of the injured limb in a model of acute joint inflammation (56) indicating the association of pain sensation and gait changes. However, in a model of chronic joint inflammation no correlation between those endpoint measurements could be observed (97). In fact, while gait alterations fully recovered, withdrawal threshold remained significantly reduced throughout the whole study (97). Habituation processes to continuous, i.e., chronic pain may be involved, enabling adequate performance of daily life activities such as walking processes (135) while mechanical allodynia persists.

#### 4.1.3. Coordination-related parameters of gait

Among the Coordination-related parameters of gait, the RI was investigated most frequently in rodent models of Monoarthritis, whereas only one study measured Phase Lag and Phase Lag Variability. While in some studies the RI remained unchanged following the induction of MA either at the knee or ankle joint (37, 47), other authors observed a significant decrement of the RI (40, 46, 68). As the RI indicates the degree of coordination, one might suggest impaired hindlimb-forelimb coordination in monoarthritic rodents regarding the decrease of this parameter. However, it needs to be considered that the general pattern generator is located within the central nervous system (71, 136) explaining why disturbed coordination has been observed after spinal cord injuries (92). A reduced RI in rodent models of MA, may have several reasons. Alterations of RI may be related to guarding behavior of the injured limb due to extensive pain perception as in several studies some rodents fully refused to use the monoarthritic limb. Subsequently, for rodents walking with 3 limbs, the RI values 0%, as the calculation requires the use of all 4 limbs during ambulation. Treatment with Rofecoxib and Morphine almost completely reversed the alterations of RI, further supporting this assumption (46). In addition to loss of paw placements, Angeby Moller et al. observed compensational extra placements of the front paws (46, 68). Both circumstances might be possible explanations for altered RI. However, changes in RI might also be attributed to detection failure of the CW software. Accordingly, the reference Paw Print necessary for determining the NSSP may be outside the recording range of the CW system and is therefore not detected. This may result in a reduced RI, as the CW might assume impaired coordination due to “missing” paw prints. However, further studies are needed, as gait analysis of patients with arthritis suggest altered intersegmental coordination (137). Chronic alterations in sensory input may influence the pattern produced by a central pattern generator (CPG) (138).

Interestingly, the injection of CARR into the knee joint, resulted in significantly altered Hind-girdle Phase Lag of rats (37), indicating that the placement of the injured limb relative to unaffected limb was significantly delayed, whereas the unaffected limb was placed prematurely. Nevertheless, Phase Lag Variability remained unchanged. Thus, adequate adaptation of coordination can be assumed, as a coordinated gait is characterized by small Phase Lag Variability (92, 139). Phase Lag Variability may therefore be a feasible parameter for the evaluation of coordination in rodent models of MA. Nevertheless, regarding Coordination-related parameters, adequate CW data analysis is fundamental, to enable valid evaluation of gait alterations.

### 4.2. Gait analysis in rodent models of polyarthritic disorders

While humans and rodents with unilateral knee OA present redistribution of weight load from the injured to the uninjured limbs mirroring the reluctance to use the arthritic limb, in bilateral OA, gait compensations were absent. Mice presented increased Duty Cycle of both hindlimbs without exhibiting any further gait alterations in contrast to unilateral injured mice (45). Concomitantly, in humans with bilateral knee OA increased Stand Time, and Double Support Time have been observed (140). However, as only one study evaluated the consequences of bilateral OA on gait behavior of mice, further studies are needed to be able to draw conclusions.

In a rat model mimicking RA, gait analysis revealed several gait alterations depicting the course of PIA (49). Pristane injection induces Polyarthritis (PA) within 2 weeks, which is followed by a phase of severe destructive arthritis. Subsequently, arthritis mitigates during a remission phase (141). In accordance, rats displayed gait modifications after 2 weeks, presenting similar to those reported for unilateral Arthritis (37, 40, 46, 47, 56, 58, 68). Rats reduced stress on the affected limbs reflected by a decrease of Print Area and Duty Cycle which was compensated by increased Print Area and Duty Cycle of the unaffected LF. However, this contrasts findings in the aforementioned model of bilateral OA, where no redistribution to the unaffected limbs could be observed (45). These changes may indicate the rodent’s demand for maintaining stability and balance during walking, when both legs are injured. Gait impairment worsened with increase of diseases severity, which is underlined by significant correlations between CW data and Arthritis Scores. After reaching a minimum, gait changes improved gradually in association with remission of PIA (49). Decrements of the Coordination-related parameter RI might be related to total loss of paw placements of the affected paws during disease progression, rather than an evident coordination disorder, as RI increased with improvements of gait behavior. In addition, gait alterations paralleled with distinct histopathological processes at the affected joints, which corresponded with disease activity and severity. Interestingly, it has to be noted that some days before macroscopic signs of arthritis such as swelling or erythema became evident, rats already displayed first gait alterations. Gait impairment paralleled with early histological signs of inflammation at the synovium and tendon sheets. We therefore assume early gait modification to be predominantly related to inflammation mediated nociception rather than altered joint mechanics due to joint destruction. It is well known that rodents as prey species try to hide and mask pain to protect in order to avoid attracting predator’s attention and becoming easy prey. Nevertheless, the CW was able to detect slight changes of gait patterns even before macroscopic signs of arthritis in the peripheral joints unveiled. These findings provide new insights into the earliest processes of PA and emphasize the presence of a pre-clinical inflammatory phase of arthritis (142) which could be addressed in future studies.

In mice models of RA, gait analysis of human tumor necrosis factor α transgenic mice (hTNFtg) and collagen-induced mice revealed comparable gait profiles to rats with PIA (38, 45, 49). A significant decrement of the General parameters Print Width, Print Length and Print Area, as well as the Pain-related parameter Max Intensity indicated pain-avoiding behavior. In the Collagen-induced Arthritis model, authors also observed significantly reduced Stride Length. Humans suffering from RA present with similar gait modifications (143). In rodents early gait changes were associated with inflammatory joint pathology (38). Previous findings in OA and MA models support the influence of inflammatory processes on early gait changes at disease onset (37, 57, 58).

Accordingly, we assume early gait changes as potential measures of inflammation-mediated pain. However, aggravation of gait disturbances over time could be observed, reflected by further decrease of gait parameters as well as occurrence of coordination impairments. In accordance with Hoffmann’s group, in the Collagen-induced model of RA an inverse correlation of gait alterations and Arthritis Score was observed – reduction of Print Area paralleled with increase in arthritis severity (42). In Hayer’s study histopathological analysis and linear regression analysis also confirmed an association between Print Length and Print Width and severe bone erosion as well as progressive cartilage degeneration (38). Therefore, at advanced diseases stages, we assume the contribution of altered joint mechanics due to progressive joint destruction on gait impairment. While treatment with an anti-TNF antibody only partially reversed gait changes, it was able to fully diminish macroscopic signs of arthritis. Thus, although macroscopic arthritis was absent rodents still demonstrated functional impairments (38). This highlights the importance of gait analysis in preclinical examination of PA.

It can be concluded that the CW gait analysis enables quantification of behavioral response correlating with disease activity and histopathological processes in rodent models of PA. The included studies support the assumption of gait as measure of pain and limb function. Subsequently, there is still not only a lot of potential for the use of the CW system in rodent models of RA as pain and functional impairments are the major symptoms of RA patients but also rodent models of rheumatological disease such as ankylosing spondylitis, gout, juvenile idiopathic arthritis or fibromyalgia.

### 4.3. Choosing the appropriate CW parameters for gait analysis in rodent models of arthritic disorders

When choosing the appropriate CW parameters for gait analysis in rodent models of arthritic disorders, one must distinguish between spatial, temporal and dynamic aspects of gait, whereas the first two groups of parameters can be subsumed under the term “spatiotemporal” (144, 145). Spatiotemporal parameters relate to the movement of an animal paws in space and time, respectively. While spatial parameters include the CW parameters subsumed under the category of General parameters of gait (I), temporal parameters relate, among others, to the Pain-related parameters (II) of Stand Time, Swing Time, Duty Cycle, Initial Contact Time, Maximum Contact Time, the Coordination-related parameters (III) of Phase Lags / Phase Dispersions, Phase Lag Variability and the Other parameters of Gait (IV) of Step Cycle, Single Stance, and Run Duration. Dynamic aspects of gait assessable via the CW include the Mean and Maximum Paw Print Intensity as well as all parameters derived from them, i.e., %TIPPI, etc.

Other authors have recommended to use a combination of spatial, e.g., Stride Length, Step Width, i.e., Base of Support and temporal parameters, e.g., Duty factor, i.e., the CW parameter Duty Cycle, and Stand Time as well as measures of footprint size, e.g., Print Area and orientation, e.g., Paw Angle, to monitor gait in rodent models of arthritic disorders (27).

According to our review’s results, alterations of spatial parameters assessable via the CW, e.g., Stride Length, Print Area occur both in rodent models of unilateral OA and MA as well as RA. Additionally, temporal parameters of gait assessable via the CW such as Duty Cycle and Stand Time reflect Pain-related changes of gait in such models of unilateral limb injury. The Pain-related parameter %TIPPI which is derived from the measured Paw Intensity also strongly correlates with the onset and relief of pain in rat models of OA and represents one of the aforementioned dynamic aspects of gait.

When choosing the appropriate parameters for gait analysis in a rodent model of an arthritic disease, one must also consider the fact whether a unilateral, i.e., monoarthritic or bilateral, i.e., polyarthritic injury is induced. Gait patterns often become asymmetric in unilateral injuries and for this purpose, in case of unilateral injuries, the analysis of Stand Time differences and Duty Cycle differences between the affected and an unaffected limb has been recommended by other authors (144). This recommendation is strongly supported by the studies included in our narrative review, in which Duty Cycle was reported among the most frequently evaluated parameters both in the Knee OA and MA group and proofed useful to monitor antalgic gait in these models.

The studies included in this review also frequently used the Pain-related parameter %TIPPI, i.e., Mean Paw Print Intensity to evaluate unilateral injury. According to Jacobs definition, this parameter is a dynamic indicator of unilateral limb injury. Given the relationship between all (Pain-related)-CW parameters assessing Paw Print Intensity and limb loading, we recommend using them as indicator for unilateral limb injury in rodent models of arthritic disorders.

Interestingly, Jacobs et al. emphasized their preference to analyze the parameter “temporal symmetry” in rodent models of unilateral arthritic disorders (144), which is best reflected by the Coordination-related CW parameter Girdle Phase Dispersions/Phase Lags/Couplings. As only one study included in our review analyzed this parameter in the context of MA future studies should elucidate whether this CW parameter should also be included among the preferred parameters to detect unilateral gait compensations.

Although less frequently studied with the CW device, bilateral arthritic disorders, require special consideration regarding the appropriate choice of gait parameters since in these kinds of injuries, the contralateral limb cannot completely compensate for the injured ipsilateral one. Therefore, the aforementioned asymmetries in regard to the General parameters of gait and Pain-related parameters of gait may be absent when comparing two affected limbs (144), which was also the case in the one study of bilateral Knee OA included in our review.

Given that rodent with bilateral limb injuries ambulate with shorter, more frequent steps, referred to as shuffle-stepping” gait (144, 145) other authors have recommended to put special emphasize on the duration of the “single-limb support” phase, which is best reflected in the CW parameter “Single Stance.” Additionally, the increased stride frequency will be reflected by a decrease in Stride Length at a given velocity, emphasizing the crucial importance to control for velocity when performing gait analysis in rodents (146). The decrease of the Coordination-related parameter RI in a rat model of RA (49) could be assumed to relate to the adaption of the aforementioned “shuffle-stepping” gait. In this regard, analysis of the Coordination-related CW parameter NSSP could be of particular interest. An analysis of NSSP has only been performed in one study of RA included in this review with insignificant results. Since significant alterations of these paw placement patterns were reported following peripheral nerve injury and are thought of a compensatory mechanism to decrease burden on the injured limb after injury, it could be interesting to investigate whether they can also be used to detect uni- or even bilateral gait compensation in rodent models of arthritic disorders.

In conclusion, when performing gait analysis in rodent models of arthritic disorders with the CW, it is highly recommended to use a set of parameters, including General Parameters (I); e.g. Stride Length, Print Area, Swing Speed, Pain-related Parameters (II); e.g. Duty Cycle, %TIPPI, Coordination-related Parameters; e.g. RI and NSSP (III) and Other Parameters of gait (IV); e.g. Single Stance, as this combination will reflect the most importance spatiotemporal and dynamic aspects of gait changes. When using a bilateral/polyarthritic model, parameters which best reflect the “shuffle stepping” gait such as Single Stance, Stride Length and the Coordination-related parameters are preferable, given the fact that unilateral compensation of the aforementioned parameters might be absent.

## 5. Further considerations for the use of the CW

We recommend conducting CW gait analysis at several time points after induction of an arthritic disorder to capture behavioral changes at different disease stages and enable differentiation between early and late alterations. Different pathogenic processes and distinct neuronal mechanisms (nociceptive sensitization, nerve fiber loss and sprouting, central sensitization, decreased descending inhibition, cortical atrophy) may be involved at different stages of disease and may lead to and define a specific individual OA pain phenotype (131–133). Further investigation of the correlation of CW data with other behavioral, histological and radiographic test, especially over time, may reveal new insights into the OA pain phenotype and underlying mechanisms.

With regard to the respective induction methods for arthritic disorders, the effects of the injections of chemical agents and the surgeries itself on gait changes must to be considered in these models as they may confound data acquisition. This may particularly affect data assessed in the first hours, days or weeks after the intervention, when rodents may also be in a state of pain due to the overall intervention, tissue trauma and local inflammatory processes. The use of sham groups may help to reveal the effect of the intervention on the data obtained. Nevertheless, ethical concerns have recently been raised about the use of sham groups alongside experimental control groups in rodent models, as the added value of a sham operation over control groups should be questioned and weighed against the need to enroll more animals in studies (147).

We highly recommend performing CW training of rodents before data acquisition. Animals should be able to perform uninterrupted, comparable runs, with minimal speed variance, which will enable reproducibility and validity of data collection (148). Several training protocols and paradigms have been published stating that animals need 1 week of training to be able to capture valid data (30, 146, 149). Moreover, it has been previously shown that velocity has a non-negligible influence on several gait parameters, leading to a multitude of speed dependent gait measures (146, 150, 151). This underlines the importance of locomotor speed control in gait analysis. Implementing standardized training protocols, supporting animals to perform runs in a specific speed range will help to overcome speed dependent hurdlers and add to the collection of reproducible, reliable and valid data (85). In this context, researchers also need to take the body weight and size dependence of specific gait parameters such as Intensity parameters into account. To reduce contribution of weight alterations on gait measures, ratio between the non-injured and injured limb can be calculated and also related to preoperative data (135). In addition, it has been shown, that gait strongly differs between rodent species and depends on strain and sex (151, 152).

To enable validity of data, comprehensive data preparation and analysis is of utmost importance. Although the CW is an automated gait system, the runs of the animals have to be checked in advance as assignments and detections errors may occur.

## 6. Conclusion

The CW gait analysis system has been widely used in the field of arthritic research as it allows for the combined monitoring of pain-related behavior and functional deficits. In this context, the CW system also enables the differentiated elicitation of gait changes in the various stages of the disease and the evaluation of therapeutic strategies. There is a great need in preclinical research to implement methods able to collect clinically relevant outcome parameters. Since the CW analysis represents an experimenter independent behavioral test enabling examination of gait during an unforced walkway crossing, the implementation of this method may ensure the assessment of clinically relevant outcomes mimicking the symptoms observed in patients. However, CW gait analysis should not be considered a stand-alone but an additional tool in the armamentarium of preclinical endpoints. Only the correlation with other established methods, such as histology, imaging modalities and other behavioral test will add to understanding of the mechanisms and processes underlying the development and progression of arthritic diseases and arthritic pain. As an automated gait analysis system, the CW allows for the assessment of a plethora of different parameters related to gait. As there is also a great variety of different models in the research field of arthritic disorder, it is of great importance to select appropriate outcome parameters that suit the particular model and purpose under investigation. This not only requires a basic understanding of the models themselves but also of the gait parameters assessable via CW.

## Author contributions

JR: Conceptualization, Methodology, Visualization, Writing – original draft, Writing – review & editing, Investigation. MM: Visualization, Writing – original draft, Writing – review & editing. SCH: Conceptualization, Writing – original draft, Writing – review & editing. TH: Conceptualization, Writing – review & editing. JK: Conceptualization, Writing – original draft, Writing – review & editing. AD: Conceptualization, Writing – review & editing. JCH: Conceptualization, Methodology, Visualization, Writing – original draft, Writing – review & editing, Investigation. CP: Conceptualization, Methodology, Writing – original draft, Writing – review & editing, Investigation.
