# Supplementary material for: Translational evaluation of gait behavior in rodent models of arthritic disorders with the CatWalk device – a narrative review
Source: Front Med (Lausanne). 2023 Oct 6;10:1255215. doi: 10.3389/fmed.2023.1255215 (PMC10587608; doi:10.3389/fmed.2023.1255215)
Supplement: Supplementary file 1 [file Table_1.pdf]

Supplementary Material

Translational evaluation of gait behavior in rodent models of arthritic disorders with the CatWalk device - a narrative review

Jana Ritter, Maximilian Menger, Steven C. Herath, Tina Histing, Jonas Kolbenschlag, Adrien Daigeler, Johannes C. Heinzel*, Cosima Prahm

*** Correspondence:** Johannes C. Heinzel: JHeinzel@bgu-tuebingen.de

1 Supplementary Tables

Supplementary Table S1. Studies evaluating Osteoarthritis by means of the CW system (n=32).

Biochemically induced Osteoarthritis (n=19)

Reference	Rodent	Joint (Side)	Induction method	Treatment	Strain	Sex	Follow up	Additional Analysis besides CW	Assessed CW parameters
Adaes 2014 (1)	rat	Knee (LH)	Collagenase	Morphine, Lidocaine, Diclofenac	W (n=99)	♂	6w	Knee-Bend Test, Knee diameter, Histopathology, Immunofluorescence for TRPV1	Total Paw Print Intensity
Adaes 2015 (2)	rat	Knee (LH)	Collagenase	Gabapentin	W (n=73)	♂	6w	Knee-Bend Test, Knee diameter, Histopathology, Immunofluorescence for ATF-3, NPY and SP, Expression of voltage-gated calcium channel subunit α2δ	Total Paw Print Intensity

Ko 2016 (3)	mice	Knee (LH)	Collagenase	HSP60	TgHSP60 (n=27), WT (n=27), FVB/N (n=48)	♂	8w	RT-PCR analysis, Immunoblotting, Histomorphometry Immunohistochemistry, μCT, Near-infrared in vivo fluorescence imaging	Max Contact Area, Max Intensity at Max Contact, Max Intensity, Print Area
Adaes 2017 (4)	rat	Knee (LH)	Collagenase	Fluorocitrate	W (n=37)	♂	6w	Knee-Bend Test, Immunohistochemical analysis of GFAP, ATF3 IBA-1, Western Blot	Total Paw Print Intensity
Ferreira-Gomes 2008 (5)	rat	Knee (LH)	MIA		W (n=?)	♂	31d	Knee-Bend Test, Pin-Prick Test, Von Frey Test, Randall-Selitto Test, Histology	Total Paw Print Intensity
Ferland 2011 (6)	rat	Knee (LH)	ACLT + pMMx or MIA	Celecoxib	SD (n=40)	♂	4w	Von Frey Test, Histology, Neuropeptide analysis of SP and CGRP	Swing Time, Swing Speed, Duty Cycle
Ferland 2012 (7)	rat	Knee (LH)	MIA	Eugenol	SD (n=23)	♂	4w	Von Frey Test, Neuropeptide analysis of SP, CGRP, Dynorphin, Histology, Toxicological assessment, Histopathology, Knee diameter	Swing Time, Swing Speed, Duty Cycle
Ferreira-Gomes 2012a (8)	rat	Knee (LH)	MIA	Lidocaine, Morphine, Diclofenac	W (n=?)	♂	20d	Histology, Knee-Bend Test	Total Paw Print Intensity
Ferreira-Gomes 2012b (9)	rat	Knee (LH)	MIA		W (n=?)	♂	31d	Knee Bend Test, Histology, Immunohistochemistry for ATF-3, NPY, GAP-43	Total Paw Print Intensity

Ishikawa 2014 (10)	rat	Knee (RH)	MIA	Diclofenac, Morphine, Tramadol, Duloxetine	SD (n=?)	♂	3w	Incapacitance Test	Max Contact Area, Swing Speed
Ishikawa 2015 (11)	rat	Knee (RH)	MIA	Anti-NGF antibody	SD (n=?)	♂	35d	ELISA based binding activities evaluation assay, Cell-based NGF function evaluation assay, Plasma concentration of Anti-NGF antibody; Knee diameter, Scoring of knee lesions	Max Contact Area, Swing Speed
Miyagi 2017 (12)	mice	Knee (RH)	MIA	Anti-NGF antibody	C57BL/6 (n=30)	♂	5w	Immunohistochemistry for CGRP	Duty Cycle, Swing Speed, Print Area
Miyamoto 2017 (13)	rat	Hip (RH)	MIA		SD (n=60)	♂	56d	Immunohistochemistry for CGRP and ATF3	21 CW parameters*
Carcole 2019 (14)	mice	Knee (?)	MIA	Sigma-1 receptor antagonist	Swiss-albino CD 1 (n=53)	♂	25d	Von Frey Test, NORT, Elevated plus maze, Elevated zero-maze test, Forced swimming test, Histology, Immunofluorescence	Print Area, Max Contact Area, Swing Time, Duty Cycle
Tschon 2020 (15)	rat	Knee (RH)	MIA	Hyaluronic acid-chitlac, Triamcinolone acetonide	SD (n=80)	♂	21d	PAM, Histology, Immunohistochemistry for type II collagen and aggrecan	Print Area, Stand Time, Swing Time, Single Stance
Caglar 2021 (16)	rat	Knee (LH)	MIA	Astaxanthin, Corticosteroid, Hyaluronic acid	W (n=44)	♂	8w ?	Histology	Print Length, Print Width, Print Area, Stride Length, Stand Time,

									Max Contact Intensity, Run Duration, Average Speed, Swing Speed
Ferreira-Gomes 2021 (17)	rat	Knee (LH)	MIA	Synthetic TLR4 Antagonist	W (n=20)	♂	28d	Knee-Bend Test, Immunohistochemical analysis for TLR4 and ATF3, Histopathology	Total Paw Print Intensity
Kara 2021 (18)	rat	Knee (LH)	MIA		W (n=10)	♂	4w	Manual walking platform for gait analysis, Histology	Print Length, Print Width, Stride Length, Average Speed, Run Duration, Maximum Contact Intensity, Print Area, Stand Time, Swing Speed
Han 2022 (19)	rat	Knee (LH)	MIA		SD (n=54?)	♂	42d	Von Frey Test, Pressure algometry	Stand Time, Print Area, Swing Speed, Duty Cycle, Single Stance, Phase Dispersion, Dynamic Weight bearing, Dynamic Weight Bearing (%), Dynamic Guarding Index

									(%)

Surgically induced Osteoarthritis (n=12)

Reference	Rodent	Joint (Side)	Induction method	Treatment	Strain	Sex	Follow up	Additional Analysis besides CW	Assessed CW parameters
Fu 2012 (20)	rat	Knee (RH)	ACLT	Buprenorphine	SD (n=18)	♀	6m	μCT, Histology	Limb Idleness Index, Target Print Ratio, Anchor Print Ratio, Swing Time
Cheuk 2016 (21)	rat	Knee (RH)	ACLT	Vitamin C + Quercetin + Deferoxamine	SD (n=12)	♀	25w	μCT, Histology	Limb Idleness Index
Siu 2019 (22)	rat	Knee (RH)	ACLT + resection of medial meniscus	DAEP (Chinese herbal paste)	SD (n=28)	♂	8w	Incapacitance Test, X-ray, Western Blot, qPCR (IL-6, TNF-α, iNOS, COX-2 and MMP-3), Quantitative analysis of the chemical markers of the DAEP and transdermal property	Stand Time, Print Area, Max Intensity, Swing Speed, Duty Cycle
Zhu 2020 (23)	mice	Knee (LH)	ACLT		C57/B6 (n=?)	♂	6m	Histology, μCT, Von Frey Test, Western Blot	Stand Time, Max Intensity at Max Intensity
Brenneis 2020 (24)	rat	Knee (RH)	ACLT + resection of medial		Lister Hooded (Crl:LIS)	♂	20w	ELISA (AGNx1 in serum), Joint diameter,	Relative Print Length (%)

			meniscus or MMT or DMM		outbred SPF (n=?)			Quantification of joint flexibility, Spontaneous activity, Histopathology, μCT	
Malfait 2010 (25)	mice	Knee (RH)	DMM	Morphine, Acetaminophen	CD-1, C57BL/6, ADAMTS-5 knockout (n=?)	♂, ♀	56d	Von Frey Test, Histology, Radiant heat paw withdrawal test, Overnight locomotor activity test	Mean Intensity at Max Contact
Muramatsu 2014 (26)	mice	Knee (LH)	DMM	Hyaluronan	C57BL/6 (n=60)	♂	16w	Histology	21 CW parameters*
Fang 2018 (27)	mice	Knee (LH)	DMM		C57/BL6 (n=?)	?	10w	μCT, Histology	Paw Intensity
Fouasson-Chailloux 2021 (28)	mice	Knee (RH) or Bilateral knees	DMM (Unilateral and bilateral)		C57BL/6 (n=24)	♂	16w	μCT, Histology	Average Speed, Print Area, Paw Print Intensity, Stand Time, Swing Time, Swing Speed, Duty Cycle
Westhof 2021 (29)	rat	Knee (RH)	DMM	Zilretta	SD (n=95)	♀	91d	Jump Incapacitance, Joint diameter	Relative Print Length (%)
Zahoor 2016 (30)	rat	Knee (LH)	Intraarticular fracture of the medial tibial plateau + internal fixation with 2		SD (n=8)	♂	8w	Histology	Initial Contact Time, Maximal Contact Time, Max Print Area, Print Area, Stride Length, Print Width, Print Length, Intensity,

			needles						Stand Time, Swing Time, Duty Cycle, Swing Speed, Paw Angle, Stand Index
Zahoor 2017 (31)	rat	Knee (LH)	Intraarticular fracture of the medial tibial plateau + internal fixation with 2 needles	Low-Intensity pulsed ultrasound	SD (n=30)	♂	8w	Histology	Initial Contact Time, Maximal Contact Time, Stride Length, Max Print Area, Print Area, Print Width, Print Length, Stand Time, Swing Time, Duty Cycle, Intensity Paw Angle Movement Vector, Swing Speed, Stand Index

LH: Left hindlimb, RH: Right hindlimb, W: Wistar, SD: Sprague Dawley, w: weeks, d: days, h: hours, m: months, TgHSP60: Heat shock protein 60 transgenic mice, WT: Wild-type , HSP60: Heat shock protein 60, TRPV1: transient receptor potential vanilloid 1, ATF-3: activating transcription factor-3, NPY: Neuropeptide Y, SP: Neuropeptide Substance P (SP), GFAP: Glial fibrillary acidic protein, IBA-1: ionized calcium-binding adapter molecule 1, MIA: Monoiodoacetate, ACLT: Anterior cruciate ligament transection, pMMx: Partial medial meniscectomy, CGRP: Calcitonin gene related peptide, GAP-43: Growth associated protein GAP-43, NGF: Nerve growth factor, NORT: Novel object recognition task, PAM: Pressure Application Measurement, TLR4: Toll-like receptor 4, IL6: Interleukin-6, TNF-α: Tumor necrosis factor α, iNOS: Inducible nitric oxide synthase, COX-2: Cyclooxygenase-2 , MMP-3: Matrix metalloproteinase- 3, MMT: Medial meniscal tear, DMM: Destabilization of medial meniscus, AGNx1: Marker of cartilage extracellular matrix remodeling, ADAMTS: A Disintegrin and Metalloproteinase with Thrombospondin Motif

*The analyzed parameters are not further specified, only significantly altered parameters are listed.

Supplementary Table S2. Studies investigating Monoarthritis via the CW system (n=9).

Reference	Rodent	Joint (Side)	Induction method	Treatment	Strain	Sex	Follow up	Additional Analysis besides CW	Assessed CW parameters
Gabriel 2007 (32)	rat	Knee (RH)	CARR		SD (n=30)	♂	48h	Von Frey Test	Mean Intensity, Duty Cycle, Base of Support, Relative Paw Placements, Max Contact Area, Print Area, Print Width, Print Length, Swing Speed, Step Cycle, Stride Length, Regularity Index, Phase Lag, Phase Lag Variability
Angeby-Moeller 2008 (33)	rat	Tibiotarsal joint (LH)	CARR	Morphine, Rofecoxib	SD (n=48)	♂	24h	None	Print Area, Mean Intensity, Duty Cycle, Regularity Index
Gabriel 2009 (34)	rat	Knee (RH)	CARR		SD (n=24)	♂	4w	Von Frey Test	Mean Intensity, Duty Cycle
Pinho 2011 (35)	rat	Tibiotarsal joint (LH)	CFA		W (n=16), SD (n=26), SHR (n=13), WKY (n=13)	♂	16d	Von Frey Test, Ankle-Bend Test, Fos expression, Inflammation score, Ankle diameter	Arthritic Paw Load (%)
Parvathy 2013 (36)	mice	Ankle (RH)	CFA	Indomethacin	C57BL/6 (n=55)	♀	7d	None	Regularity Index, Mean Intensity at Max Contact, Print

									Area, Stand Time, Stride Length, Swing Time, Duty Cycle, Swing Speed
Angeby Moeller 2018 (37)	rat	Tibiotarsal joint (LH)	CFA	Naproxen, Pregabalin	W (n=104)	♂	4d	Incapacitance Test, Von Frey Test	Regularity Index, Walking Speed, Duty Cycle, Dynamic Weight Bearing, Dynamic Weight Bearing (%), Dynamic Guarding Index (%)
Angeby Moeller 2020 (38)	mice	OA: Knee (LH); MA: Ankle (LH)	OA: ACLT, MA: CFA	Anti-NGF antibody	C57BL/6JRj (n=181)	♀	14d	Histopathology, Stationary weight bearing, Ankle joint swelling	Regularity Index, Walking Speed, Duty Cycle, Swing Speed, Stride Length, Weight bearing, Weight bearing (%), Guarding index (%)
Masocha 2009 (39)	mice	Ankle (RH)	LPS	Indomethacin	C57BL/6 (n=71)	?	7d	None	Mean Intensity at Max Contact, Print Area, Regularity Index
Abu-Ghefreh 2010 (40)	mice	Ankle (RH)	LPS	Minocycline, Indomethacin, Selective COX1 inhibitor, Selective COX 2 Inhibitor	BALB/c (n=338), C57BL/6 (n=32)	?	2d	Hot Plate Test	Mean Intensity at Max Contact, Print Area

CARR: Carrageenan, CFA: Complete Freund’s adjuvant, LPS: Lipopolysaccharide, LH: Left hindlimb, RH: Right hindlimb, W: Wistar, SD: Sprague Dawley, w: weeks, d: days, h: hours, SHR: Spontaneously hypertensive rats, WKY: normotensive Wistar Kyoto rat, OA: Osteoarthritis, MA: Monoarthritis, NGF: Nerve growth factor, ACLT: Anterior cruciate ligament transection

Supplementary Table S3. Studies investigating polyarthritic disorders via the CW system (n=3).

Reference	Rodent	Type of injury	Induction method	Treatment	Strain	Sex	Follow up	Additional Analysis besides CW	Assessed CW parameters
Hayer 2016 (41)	mice	Rheumatoid Arthritis	hTNFtg mice	Anti-TNF antibody (Infliximab)	hTNFtg, WT (n=?)	♀	Week 5 after birth until end of week 15 after birth	Paw Swelling, Grip strength, μ-CT, Histopathology	Print Area, Print Length, Print Width, Max Contact Area, Stride Length, Swing Speed, Max Intensity, Mean Intensity, Regularity Index, Phase Dispersion, Print Positions
Mausset-Bonnefont 2019 (42)	mice	Rheumatoid Arthritis	Collagen type II	Methotrexate	DBA/1OlaHsd (n=91)	♂	49d	Open Field Test, Von Frey Test, Hargreaves Test, Static weight-bearing, Skin Temperature, μ-CT, Histopathology, Paw swelling, Clinical Arthritis Scoring	Print Area, Max Intensity, Stand Time, Stride Length, Run Duration
Hoffman 2010 (43)	rat	Polyarthritis	Pristane		DA (n=?)	♂/♀	35d	Histology, Histomorphometry	Print Area, Stand Time, Regularity Index

WT: Wild-type, d: days, TNF: Tumor necrosis factor, hTNFtg: human tumor necrosis factor α transgenic mice, DA: Dark Agouti

Bibliography

1. Adaes S, Mendonca M, Santos TN, Castro-Lopes JM, Ferreira-Gomes J, Neto FL. Intra-Articular Injection of Collagenase in the Knee of Rats as an Alternative Model to Study Nociception Associated with Osteoarthritis. *Arthritis Res Ther* (2014) 16(1):R10. Epub 2014/01/16. doi: 10.1186/ar4436.
2. Adaes S, Ferreira-Gomes J, Mendonca M, Almeida L, Castro-Lopes JM, Neto FL. Injury of Primary Afferent Neurons May Contribute to Osteoarthritis Induced Pain: An Experimental Study Using the Collagenase Model in Rats. *Osteoarthritis Cartilage* (2015) 23(6):914-24. Epub 2015/02/24. doi: 10.1016/j.joca.2015.02.010.
3. Ko JY, Sun YC, Li WC, Wang FS. Chaperonin 60 Regulation of Sox9 Ubiquitination Mitigates the Development of Knee Osteoarthritis. *J Mol Med (Berl)* (2016) 94(7):755-69. Epub 20160427. doi: 10.1007/s00109-016-1422-3.
4. Adaes S, Almeida L, Potes CS, Ferreira AR, Castro-Lopes JM, Ferreira-Gomes J, et al. Glial Activation in the Collagenase Model of Nociception Associated with Osteoarthritis. *Mol Pain* (2017) 13:1744806916688219. Epub 2017/03/23. doi: 10.1177/1744806916688219.
5. Ferreira-Gomes J, Adaes S, Castro-Lopes JM. Assessment of Movement-Evoked Pain in Osteoarthritis by the Knee-Bend and Catwalk Tests: A Clinically Relevant Study. *J Pain* (2008) 9(10):945-54. Epub 2008/07/25. doi: 10.1016/j.jpain.2008.05.012.
6. Ferland CE, Laverty S, Beaudry F, Vachon P. Gait Analysis and Pain Response of Two Rodent Models of Osteoarthritis. *Pharmacol Biochem Behav* (2011) 97(3):603-10. Epub 2010/11/30. doi: 10.1016/j.pbb.2010.11.003.
7. Ferland CE, Beaudry F, Vachon P. Antinociceptive Effects of Eugenol Evaluated in a Monoiodoacetate-Induced Osteoarthritis Rat Model. *Phytother Res* (2012) 26(9):1278-85. Epub 2012/09/28. doi: 10.1002/ptr.3725.
8. Ferreira-Gomes J, Adaes S, Mendonca M, Castro-Lopes JM. Analgesic Effects of Lidocaine, Morphine and Diclofenac on Movement-Induced Nociception, as Assessed by the Knee-Bend and Catwalk Tests in a Rat Model of Osteoarthritis. *Pharmacol Biochem Behav* (2012) 101(4):617-24. Epub 2012/03/20. doi: 10.1016/j.pbb.2012.03.003.
9. Ferreira-Gomes J, Adaes S, Sousa RM, Mendonca M, Castro-Lopes JM. Dose-Dependent Expression of Neuronal Injury Markers During Experimental Osteoarthritis Induced by Monoiodoacetate in the Rat. *Mol Pain* (2012) 8:50. Epub 2012/07/10. doi: 10.1186/1744-8069-8-50.
10. Ishikawa G, Nagakura Y, Takeshita N, Shimizu Y. Efficacy of Drugs with Different Mechanisms of Action in Relieving Spontaneous Pain at Rest and During Movement in a Rat Model of Osteoarthritis. *Eur J Pharmacol* (2014) 738:111-7. Epub 2014/06/19. doi: 10.1016/j.ejphar.2014.05.048.
11. Ishikawa G, Koya Y, Tanaka H, Nagakura Y. Long-Term Analgesic Effect of a Single Dose of Anti-Ngf Antibody on Pain During Motion without Notable Suppression of Joint Edema and Lesion in a Rat Model of Osteoarthritis. *Osteoarthritis Cartilage* (2015) 23(6):925-32. Epub 2015/02/14. doi: 10.1016/j.joca.2015.02.002.
12. Miyagi M, Ishikawa T, Kamoda H, Suzuki M, Inoue G, Sakuma Y, et al. Efficacy of Nerve Growth Factor Antibody in a Knee Osteoarthritis Pain Model in Mice. *BMC Musculoskelet Disord* (2017) 18(1):428. Epub 2017/11/05. doi: 10.1186/s12891-017-1792-x.

13. Miyamoto S, Nakamura J, Ohtori S, Orita S, Nakajima T, Omae T, et al. Pain-Related Behavior and the Characteristics of Dorsal-Root Ganglia in a Rat Model of Hip Osteoarthritis Induced by Mono-Iodoacetate. *J Orthop Res* (2017) 35(7):1424-30. Epub 2016/08/21. doi: 10.1002/jor.23395.
14. Carcole M, Zamanillo D, Merlos M, Fernandez-Pastor B, Cabanero D, Maldonado R. Blockade of the Sigma-1 Receptor Relieves Cognitive and Emotional Impairments Associated to Chronic Osteoarthritis Pain. *Front Pharmacol* (2019) 10:468. Epub 2019/05/28. doi: 10.3389/fphar.2019.00468.
15. Tschon M, Salamanna F, Martini L, Giavaresi G, Lorenzini L, Calza L, et al. Boosting the Intra-Articular Efficacy of Low Dose Corticosteroid through a Biopolymeric Matrix: An in Vivo Model of Osteoarthritis. *Cells* (2020) 9(7). Epub 2020/07/02. doi: 10.3390/cells9071571.
16. Çağlar C, Kara H, Ateş O, Uğurlu M. Evaluation of Different Intraarticular Injection Therapies with Gait Analysis in a Rat Osteoarthritis Model. *Cartilage* (2021) 13(2_suppl):1134s-43s. Epub 2021/09/17. doi: 10.1177/19476035211046042.
17. Ferreira-Gomes J, Garcia MM, Nascimento D, Almeida L, Quesada E, Castro-Lopes JM, et al. Tlr4 Antagonism Reduces Movement-Induced Nociception and Atf-3 Expression in Experimental Osteoarthritis. *J Pain Res* (2021) 14:2615-27. Epub 20210824. doi: 10.2147/jpr.S317877.
18. Kara H, Çağlar C, Asiltürk M, Karahan S, Uğurlu M. Comparison of a Manual Walking Platform and the Catwalk Gait Analysis System in a Rat Osteoarthritis Model. *Adv Clin Exp Med* (2021) 30(9):949-56. Epub 2021/08/14. doi: 10.17219/acem/137536.
19. Han FY, Brockman DA, Nicholson JR, Corradini L, Smith MT. Gait Analysis as a Robust Pain Behavioural Endpoint in the Chronic Phase of the Monoiodoacetate-Induced Knee Joint Pain in the Rat. *Behav Pharmacol* (2022) 33(1):23-31. doi: 10.1097/fbp.0000000000000663.
20. Fu SC, Cheuk YC, Hung LK, Chan KM. Limb Idleness Index (Lii): A Novel Measurement of Pain in a Rat Model of Osteoarthritis. *Osteoarthritis Cartilage* (2012) 20(11):1409-16. Epub 2012/08/15. doi: 10.1016/j.joca.2012.08.006.
21. Cheuk YC, Fu SC, Mok SW, Ho KK, Hung LK, Chan KM. Intra-Articular Injection of an Antioxidant Formulation Did Not Improve Structural Degeneration in a Rat Model of Post-Traumatic Osteoarthritis. *J Orthop Translat* (2017) 8:25-31. Epub 2016/09/08. doi: 10.1016/j.jot.2016.08.001.
22. Siu WS, Shum WT, Cheng W, Wong CW, Shiu HT, Ko CH, et al. Topical Application of Chinese Herbal Medicine Daep Relieves the Osteoarthritic Knee Pain in Rats. *Chin Med* (2019) 14:55. Epub 2019/12/13. doi: 10.1186/s13020-019-0278-1.
23. Zhu J, Zhu Y, Xiao W, Hu Y, Li Y. Instability and Excessive Mechanical Loading Mediate Subchondral Bone Changes to Induce Osteoarthritis. *Ann Transl Med* (2020) 8(6):350. Epub 2020/05/02. doi: 10.21037/atm.2020.02.103.
24. Brenneis C, Menges S, Westhof A, Lindemann S, Thudium CS, Kleinschmidt-Doerr K. Colony Housing Promotes Structural and Functional Changes During Surgically Induced Osteoarthritis in Rats. *Osteoarthr Cartil Open* (2020) 2(4):100100. Epub 2020/09/16. doi: 10.1016/j.ocarto.2020.100100.
25. Malfait AM, Ritchie J, Gil AS, Austin JS, Hartke J, Qin W, et al. Adamts-5 Deficient Mice Do Not Develop Mechanical Allodynia Associated with Osteoarthritis Following Medial Meniscal Destabilization. *Osteoarthritis Cartilage* (2010) 18(4):572-80. Epub 2009/12/29. doi: 10.1016/j.joca.2009.11.013.

26. Muramatsu Y, Sasho T, Saito M, Yamaguchi S, Akagi R, Mukoyama S, et al. Preventive Effects of Hyaluronan from Deterioration of Gait Parameters in Surgically Induced Mice Osteoarthritic Knee Model. *Osteoarthritis Cartilage* (2014) 22(6):831-5. Epub 20140403. doi: 10.1016/j.joca.2014.03.016.
27. Fang H, Huang L, Welch I, Norley C, Holdsworth DW, Beier F, et al. Early Changes of Articular Cartilage and Subchondral Bone in the Dmm Mouse Model of Osteoarthritis. *Sci Rep* (2018) 8(1):2855. Epub 2018/02/13. doi: 10.1038/s41598-018-21184-5.
28. Fouasson-Chailloux A, Dauty M, Bodic B, Masson M, Maugars Y, Metayer B, et al. Posttraumatic Osteoarthritis Damage in Mice: From Histological and Micro-Computed Tomodensitometric Changes to Gait Disturbance. *Cartilage* (2021) 13(2_suppl):1478s-89s. Epub 2021/10/27. doi: 10.1177/19476035211053821.
29. Westhof A, Kleinschmidt-Doerr K, Michaelis M, Brenneis C. Dynamic Weight-Bearing Test During Jumping: A Sensitive Outcome Measure of Chronic Osteoarthritis Pain in Rats. *Heliyon* (2021) 7(9):e07906. Epub 2021/09/16. doi: 10.1016/j.heliyon.2021.e07906.
30. Zahoor T, Mitchell R, Bhasin P, Schon L, Zhang Z. A Surgical Model of Posttraumatic Osteoarthritis with Histological and Gait Validation. *Orthop J Sports Med* (2016) 4(7):2325967116658874. Epub 20160728. doi: 10.1177/2325967116658874.
31. Zahoor T, Mitchell R, Bhasin P, Guo Y, Paudel S, Schon L, et al. Effect of Low-Intensity Pulsed Ultrasound on Joint Injury and Post-Traumatic Osteoarthritis: An Animal Study. *Ultrasound Med Biol* (2018) 44(1):234-42. Epub 20171027. doi: 10.1016/j.ultrasmedbio.2017.09.014.
32. Gabriel AF, Marcus MA, Honig WM, Walenkamp GH, Joosten EA. The Catwalk Method: A Detailed Analysis of Behavioral Changes after Acute Inflammatory Pain in the Rat. *J Neurosci Methods* (2007) 163(1):9-16. Epub 2007/03/27. doi: 10.1016/j.jneumeth.2007.02.003.
33. Angeby Moller K, Berge O-G, Hamers FPT. Using the Catwalk Method to Assess Weight-Bearing and Pain Behaviour in Walking Rats with Ankle Joint Monoarthritis Induced by Carrageenan: Effects of Morphine and Rofecoxib. *J Neurosci Methods* (2008) 174(1):1-9. Epub 2008/07/19. doi: 10.1016/j.jneumeth.2008.06.017.
34. Gabriel AF, Marcus MA, Walenkamp GH, Joosten EA. The Catwalk Method: Assessment of Mechanical Allodynia in Experimental Chronic Pain. *Behav Brain Res* (2009) 198(2):477-80. Epub 2009/01/17. doi: 10.1016/j.bbr.2008.12.018.
35. Pinho D, Morato M, Couto MR, Marques-Lopes J, Tavares I, Albino-Teixeira A. Does Chronic Pain Alter the Normal Interaction between Cardiovascular and Pain Regulatory Systems? Pain Modulation in the Hypertensive-Monoarthritic Rat. *J Pain* (2011) 12(2):194-204. Epub 2010/08/26. doi: 10.1016/j.jpain.2010.06.009.
36. Parvathy SS, Masocha W. Gait Analysis of C57bl/6 Mice with Complete Freund's Adjuvant-Induced Arthritis Using the Catwalk System. *BMC Musculoskelet Disord* (2013) 14:14. Epub 2013/01/10. doi: 10.1186/1471-2474-14-14.
37. Angeby Moller K, Svard H, Suominen A, Immonen J, Holappa J, Stenfors C. Gait Analysis and Weight Bearing in Pre-Clinical Joint Pain Research. *J Neurosci Methods* (2018) 300:92-102. Epub 2017/04/27. doi: 10.1016/j.jneumeth.2017.04.011.
38. Angeby Moller K, Aulin C, Baharpoor A, Svensson CI. Pain Behaviour Assessments by Gait and Weight Bearing in Surgically Induced Osteoarthritis and Inflammatory Arthritis. *Physiol Behav* (2020) 225:113079. Epub 2020/07/18. doi: 10.1016/j.physbeh.2020.113079.

39. Masocha W, Parvathy SS. Assessment of Weight Bearing Changes and Pharmacological Antinociception in Mice with Lps-Induced Monoarthritis Using the Catwalk Gait Analysis System. *Life Sci* (2009) 85(11-12):462-9. Epub 2009/08/18. doi: 10.1016/j.lfs.2009.07.015.
40. Abu-Ghefreh AA, Masocha W. Enhancement of Antinociception by Coadministration of Minocycline and a Non-Steroidal Anti-Inflammatory Drug Indomethacin in Naïve Mice and Murine Models of Lps-Induced Thermal Hyperalgesia and Monoarthritis. *BMC Musculoskelet Disord* (2010) 11:276. Epub 20101201. doi: 10.1186/1471-2474-11-276.
41. Hayer S, Bauer G, Willburger M, Sinn K, Alasti F, Plasenzotti R, et al. Cartilage Damage and Bone Erosion Are More Prominent Determinants of Functional Impairment in Longstanding Experimental Arthritis Than Synovial Inflammation. *Dis Model Mech* (2016) 9(11):1329-38. Epub 2016/09/18. doi: 10.1242/dmm.025460.
42. Mausset-Bonnefont AL, Cren M, Vicente R, Quentin J, Jorgensen C, Apparailly F, et al. Arthritis Sensory and Motor Scale: Predicting Functional Deficits from the Clinical Score in Collagen-Induced Arthritis. *Arthritis Res Ther* (2019) 21(1):264. Epub 2019/12/06. doi: 10.1186/s13075-019-2047-z.
43. Hoffmann MH, Hopf R, Niederreiter B, Redl H, Smolen JS, Steiner G. Gait Changes Precede Overt Arthritis and Strongly Correlate with Symptoms and Histopathological Events in Pristane-Induced Arthritis. *Arthritis Res Ther* (2010) 12(2):R41. Epub 2010/03/13. doi: 10.1186/ar2950.
